# Affinity-based protein profiling of MDM2 inhibitor Navtemadlin†

**DOI:** 10.1039/d5sc00120j

**Published:** 2025-03-12

**Authors:** Amrita Date, Archie Wall, Peiyu Zhang, Jack W. Houghton, Jianan Lu, Adam M. Thomas, Tristan Kovačič, Andrew J. Wilson, Edward W. Tate, Anna Barnard

**Affiliations:** a Department of Chemistry, Molecular Sciences Research Hub, Imperial College London 82 Wood Lane London W12 0BZ UK a.barnard@imperial.ac.uk; b Astbury Centre for Structural Molecular Biology, University of Leeds Woodhouse Lane Leeds LS2 9JT UK; c School of Chemistry, University of Leeds Woodhouse Lane Leeds LS2 9JT UK; d School of Chemistry, University of Birmingham Edgbaston Birmingham B15 2TT UK; e The Francis Crick Institute London NW1 1AT UK

## Abstract

Navtemadlin is a potent inhibitor of the p53-MDM2 protein–protein interaction, which plays a critical role in the proliferation of p53-wildtype tumours. Whilst Navtemadlin has progressed to multiple Phase III clinical trials in oncology, little has been disclosed regarding its selectivity for MDM2 in cells. Here, we report the synthesis and validation of photoactivatable clickable probes of Navtemadlin, and their application to *de novo* target discovery for Navtemadlin through affinity-based protein profiling. MDM2 was robustly identified as the main target, across two cell lines, using two distinct probe designs. While off-targets were identified, these were not consistent across cell lines and probe designs, consistent with a high degree of selectivity for the target protein. Whole proteome profiling experiments across different time points confirmed p53-mediated phenotypic activity and revealed novel expression patterns for key proteins in the p53 pathway.

## Abstract 2

Affinity-based protein profiling of Navtemadlin, a potent MDM2 inhibitor, revealed exceptional selectivity for the target protein in two p53-wildtype cell lines, using two structurally distinct photoaffinity probes.

## Graphical abstract

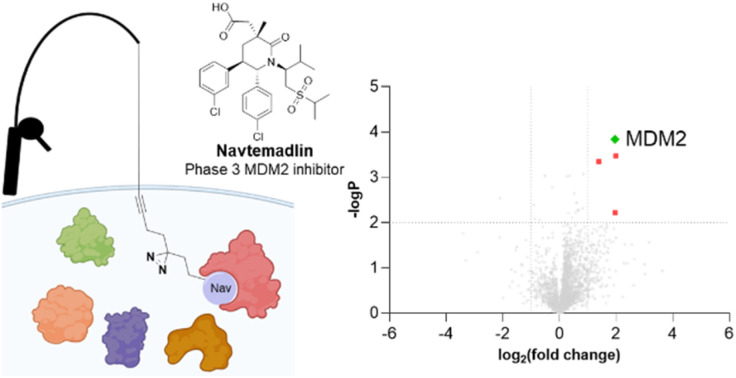

The association of murine double minute 2 (MDM2) and the tumour suppressor protein, p53 is among the most studied protein–protein interactions (PPIs) in oncology. The activity of p53 in healthy cells is tightly regulated. However, in response to cellular stresses, such as DNA damage or hypoxia, which can lead to tumour progression, p53 is activated. It acts as a transcription factor, binding to DNA to induce the expression of proteins involved in cell cycle arrest, apoptosis, and DNA repair, while suppressing transcription of cell survival genes, such as BCL-2 and MCL-1.^1,2^ The importance of p53 in preventing the progression of tumours is well documented; the gene is found to be either inactive or mutated in approximately 50% of human cancers.^3–6^ In cases where wildtype p53 is present, its activity is suppressed through various signalling pathways,^7^ most notably through the action of MDM2, its key negative regulator. In healthy cells, MDM2 function is critical for cell survival, however, if overexpressed in cancer cells, it impedes the tumour suppressor function of p53.^8^ It achieves this in two ways: firstly by binding to the transactivation domain of p53, obstructing its ability to bind to DNA, and secondly through its E3 ligase activity, facilitating the ubiquitination and proteasomal degradation of p53.^8^ Inhibition of MDM2, through a range of approaches, can lead to restoration of p53 function and slow processes associated with tumour progression, making it a promising therapeutic strategy in the treatment of cancer. This has been widely explored through competitive inhibition of the MDM2-p53 interaction, MDM2 degradation or destabilisation, and inhibition of MDM2 E3 ligase activity.^1–3,9,10^ However, despite decades of research and tens of candidates entering clinical trials, no MDM2 inhibitors have yet been approved for clinical use.^10^

Navtemadlin (KRT-232, formerly AMG-232) is a potent competitive inhibitor of the p53-MDM2 PPI. Originally developed by Amgen through a structure-based drug design approach, this piperidinone-based molecule is currently being tested in three Phase III, and several more Phase I/II clinical trials by Kartos Therapeutics.^11–13^ Navtemadlin has been reported to exhibit sub-nanomolar binding to MDM2 in biophysical and biochemical assays.^11^ Promising clinical results have been observed for the treatment of metastatic cutaneous melanoma and Merkle cell carcinoma, and trials are ongoing to assess its value in the treatment of myelofibrosis.^14^ While gastrointestinal and haematological adverse effects have been observed, the overall safety profile has been deemed favourable.^15,16^ Despite progress in the clinic, knowledge of the selectivity and off-targets of Navtemadlin remains limited. Reports of its selectivity have thus far hinged on its lack of activity in p53 knockout systems.^11,17^ While such approaches are valuable in confirming drug targets, they do not account for unexpected phenotypic responses arising as a result of potential off-target engagement.

Mass spectrometry-based proteomics offers a global approach for target identification, enabling *de novo* elucidation of the target profile of a molecule within a proteome. This can offer an insight into the selectivity of a molecule, help determine off-target binding which may lead to adverse drug reactions, and facilitate analysis of pathways responsible for downstream effects.^18,19^ With rapid developments in proteomics instrumentation and data analysis over the last five years, exceptional depth of proteome coverage can now be achieved.^20^ Here, we report a proteome-wide affinity-based protein profiling (ABPP) approach to probe the cellular selectivity and target profile of Navtemadlin, using a next-generation proteomics platform. Our approach uses a diazirine-based photoactivatable clickable probe which retains the binding properties and phenotypic activity of Navtemadlin, whilst imbuing it with the capacity to covalently label proteins in its proximity upon irradiation with ultraviolet light.^21^ A diazirine-based approach was chosen for its small size and ability to achieve indiscriminate insertion into various covalent bonds, compared to other labelling strategies previously used to study MDM2 inhibitors.^22–24^

Fully functionalised probe (FFP) design for Navtemadlin was guided by the ligand-bound co-crystal structure of MDM2 (PDB:4JRG).^11^ Docking Navtemadlin into this crystal structure revealed the availability of the ring methyl substituent, carboxylic acid, and isopropyl sulfone moieties for modification, without disrupting key binding interactions (Fig. 1A). Based on the synthetic accessibility and feasibility of late stage diazirine incorporation, the free carboxylic acid was chosen for modification. Probes 1 and 2 were synthesised by introduction of a photoactivatable diazirine group and an alkyne handle through HATU-mediated amide coupling reactions (Fig. 1B). Despite its simple structure, we were wary of the impact that the minimalist tag would have on the effectiveness of the FFP,^25^ leading to incorporation of two different tag designs. Probe 1 incorporated a linear tag, while probe 2 contained a branched tag, with both probes obtained in high purity (>99%).

**Fig. 1:**
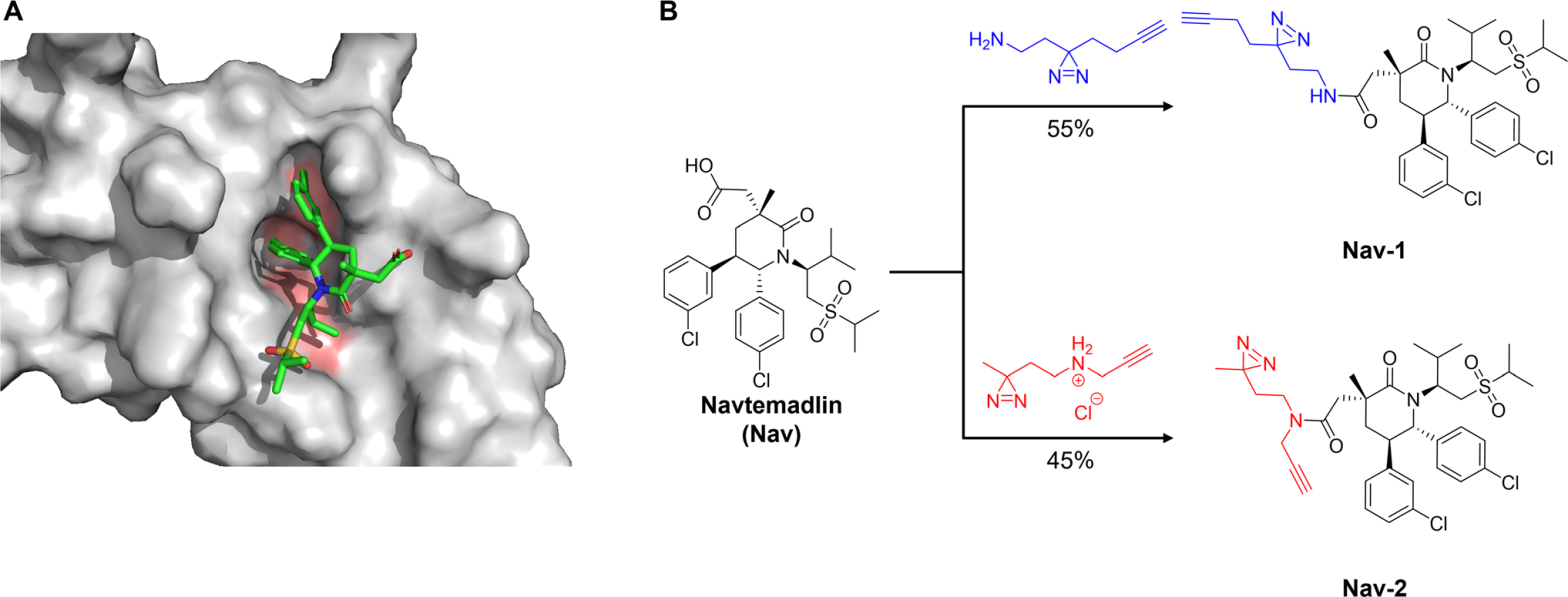
Design and synthesis of photoactivatable clickable probe of Navtemadlin. (A) Navtemadlin docked into crystal structure of MDM2 (PDB:4JRG, docked using Molecular Operating Environment), displaying the availability of the carboxylic acid functionality for modification. Key binding pockets on MDM2 are highlighted in orange. (B) Synthesis of probes 1 and 2 from Navtemadlin. Reaction was conducted in presence of HATU and DIPEA in DMF at room temperature overnight.

We next sought to confirm probe binding to MDM2 and retention of phenotypic activity in cells. MDM2 binding was assessed by means of a fluorescence anisotropy competition binding assay, using a fluorescein-labelled p53 peptide as a tracer.^26^ This showed a 4-fold and 10-fold drop in binding affinity compared to Navtemadlin for probes 1 and 2, respectively (Fig. 2A, B and S1†). Despite this, both probes maintained sub-micromolar binding affinities, making them suitable for use in in-cell experiments. Probe activity was then evaluated in two p53-wildtype cell lines: SJSA-1, which overexpresses MDM2, and MCF-7, which shows lower MDM2 expression but overexpresses MDM2 homolog murine double minute *X* (MDM*X*) (Fig. S2†). In-cell p53-mediated activity upon probe treatment was monitored by observation of the expression of p53-downstream proteins, p21 and MDM2, which both increased upon probe treatment, with the concentrations required to achieve this effect being approximately 5-fold and 10-fold higher for 1 and 2, respectively, than required for Navtemadlin; this was consistent with their relative differences in binding affinity (Fig. 2C). The effect of probe treatment on cell viability was assessed through a colourimetric assay using 3-(4,5-dimethylthiazol-2-yl)-5-(3-carboxymethoxyphenyl)-2-(4-sulfophenyl)-2*H*-tetrazolium (MTS) reagent. This revealed attenuation of activity of the probes compared to Navtemadlin in both SJSA-1 and MCF-7 cell lines (Fig. 2B and S3†), which was consistent with the observed decrease in MDM2 binding affinity. To further understand the activity of these molecules, cell proliferation and cytotoxicity were monitored by real-time live cell imaging over five days, with fluorescent signal from SYTOX green nucleic acid stain used to assess cell death and confluence measured based on phase-contrast imaging. Both probes showed similar behaviour in the SJSA-1 cell line to Navtemadlin but required a 5-fold higher concentration to achieve comparable cytotoxic activity (Fig. 2D(i) and (iii)). Navtemadlin is reported to induce cell death in cell lines overexpressing MDM2, while only inhibition of cell proliferation has been observed in those with lower MDM2 expression.^27^ As a reflection of this, cytostatic rather than cytotoxic effects were observed in the MCF-7 cell line (Fig. 2D(ii) and (iv)).

**Fig. 2:**
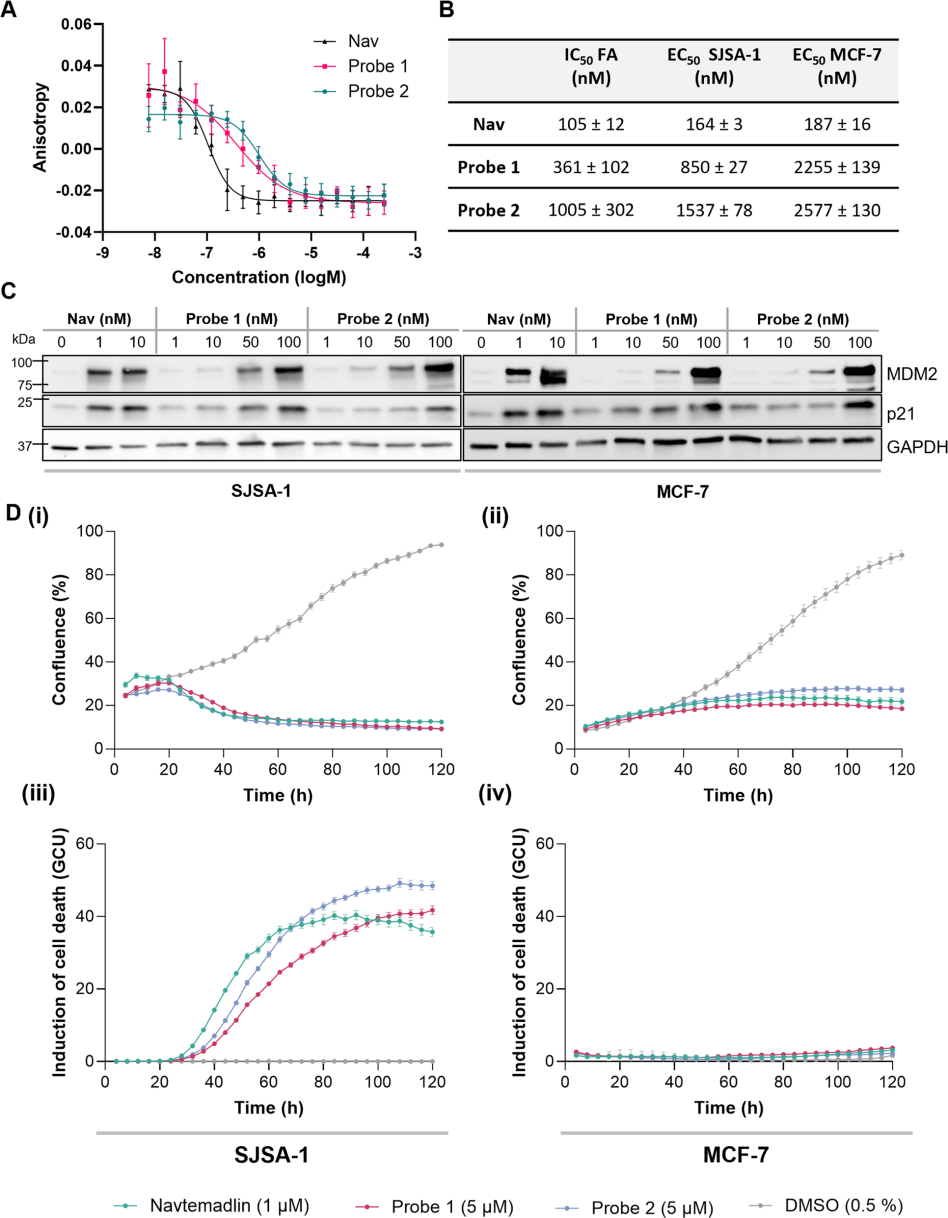
Validation of binding and phenotypic activity of probes. (A) Quantification of binding affinity through a fluorescence anisotropy competition assay. (B) IC_50_ values obtained from fluorescence anisotropy assay (*n* = 3) and EC_50_ values from MTS assay following 72 h treatment (*n* = 3). (C) Western blot showing the upregulation of p21 and MDM2 in SJSA-1 and MCF-7 cell lines, upon treatment with Navtemadlin and probes. (D) Real-time live cell imaging data, showing the effect of Navtemadlin/probe treatment on confluence (i and ii) and cytotoxicity (plotted as a ratio of the total intensity of the fluorescent response from SYTOX green per well to the area covered by cells; (iii and iv) in SJSA-1 and MCF-7 cell lines, recorded over five days using an IncuCyte cell imager (mean ± SEM, *n* = 3).

Next, validation of UV-induced diazirine-mediated protein labelling by the probes was carried out initially using recombinant MDM2, which was treated with the probe and irradiated for 10 min at 365 nm. The efficiency of protein labelling was determined through intact protein mass spectrometry, which indicated approximately 50% labelling with probe 1 and 18% labelling with probe 2 (Fig. 3A). In both cases, labelling was reduced to less than 5% in the presence of a 10-fold excess of Navtemadlin. The availability of the alkyne handle for copper-catalysed alkyne–azide cycloaddition (CuAAC) after protein labelling was assessed by reaction of the protein-bound probe with Calfluor647 azide and resolution of the protein by SDS-PAGE. Where protein labelling was achieved, a fluorescent band was observed on the gel (Fig. S4†). To appraise in-cell MDM2 labelling, SJSA-1 and MCF-7 cell lines were treated with the probes for 4 h and then irradiated at 365 nm. Cells were subsequently lysed, and CuAAC ligation carried out with azide-TAMRA-biotin (AzTB). Labelled proteins were enriched onto Neutravidin agarose resin and visualised by western blot. Since treatment with MDM2 inhibitors upregulates MDM2, treatment conditions were optimised such that Navtemadlin-treated cells, used as the negative control, showed similar levels of MDM2 expression as the probe-treated cells, confirmed by western blot and proteomics (Fig. S5†). Light-dependant MDM2 labelling was seen for both probes in an SJSA-1 cell line (Fig. 3B and S6†). Probe treatment in the presence of an excess of Navtemadlin resulted in an effective reduction in labelling (“off-compete”), which was observed as a reduction in the intensity of the enrichment band. Unsurprisingly, MDM2 labelling was significantly more difficult to observe in an MCF-7 cell line, given its inherently low abundance. Some labelling was observed in samples treated with probe 2, whilst this was lower with probe 1 (Fig. 3B and S6†). Overall, these validation experiments demonstrate that the probes retain binding affinity for MDM2, exhibit similar phenotypic activity to Navtemadlin, and covalently label the target protein in cells, confirming their suitability for use in affinity-based protein profiling experiments.

**Fig. 3:**
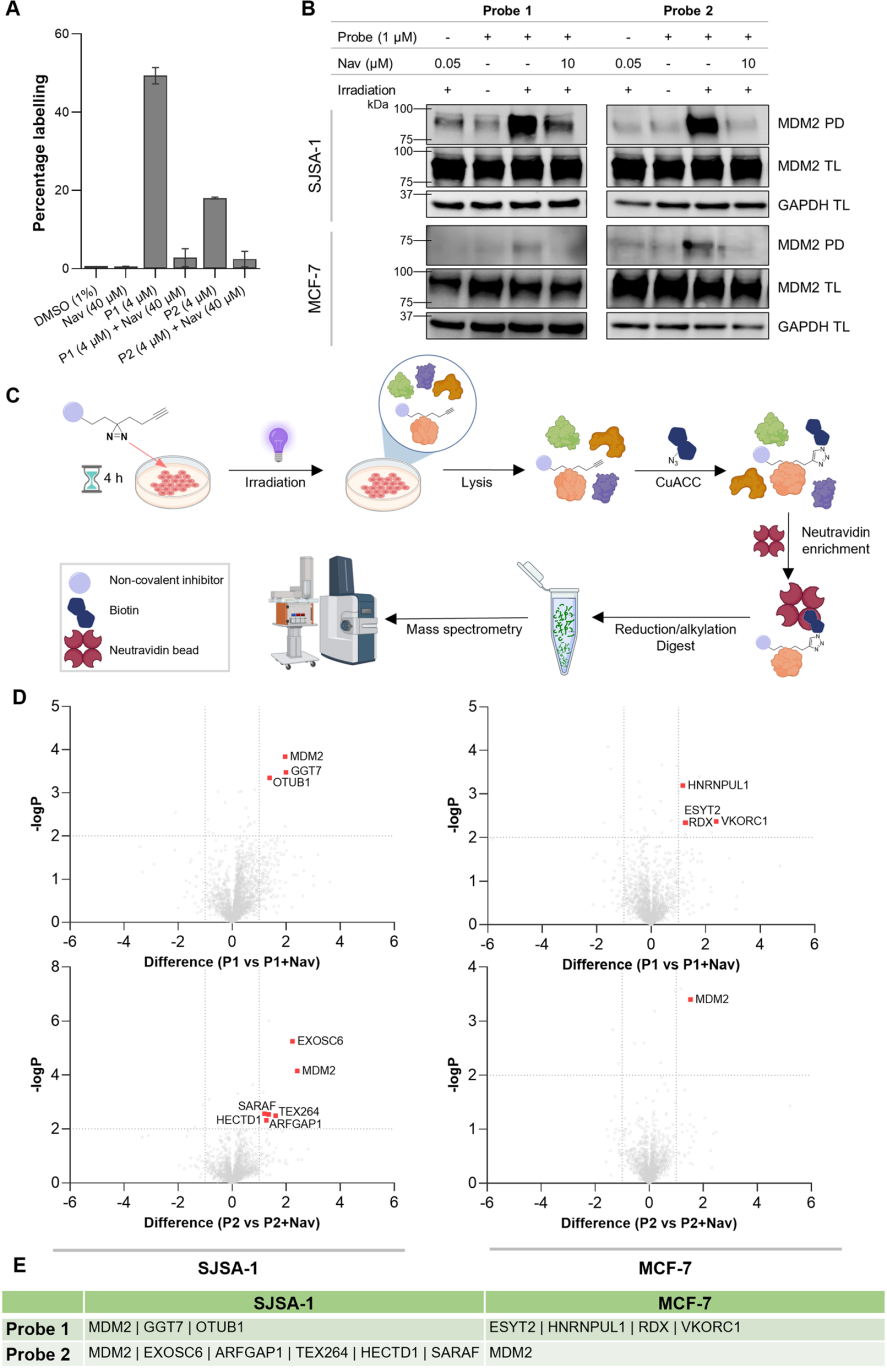
Validation of covalent protein labelling by probes and application of probes in affinity-based protein profiling experiments. (A) Covalent labelling of recombinant MDM2 quantified through intact protein mass spectrometry measurements (*n* = 3). (B) Western blot showing the light-dependant labelling and pulldown (PD) of MDM2 using FFPs in SJSA-1 and MCF-7 cell lines. Western blots of total lysate (TL) showing comparable input across conditions. (C) Summary of affinity-based protein profiling workflow. (D) Target engagement profiles of probe 1 and probe 2 in SJSA-1 and MCF-7 cell lines. Volcano plots showing differences in enrichment (*x*-axis) between live cells treated with 1 μM probe (right) *versus* 1 μM probe + 10 μM Navtemadlin (left). Associated significance (*y*-axis) is determined by paired Student's *t*-test (FDR = 0.05, *S*_0_ = 0.1, *n* = 4). Statistically significant hits identified are highlighted in red. Total proteins quantified = 2127 in SJSA-1 and 1797 in MCF-7. (E) List of significant hits identified from affinity-based protein profiling experiments.

The validated probes were then applied in chemical proteomics experiments to understand the selectivity and target profile of Navtemadlin. Here, cells were treated with the probes, irradiated, and lysed. CuAAC ligation was carried out with azide-PEG_3_-biotin (AzB), labelled proteins were enriched on Neutravidin agarose resin and subjected to reduction and alkylation, and digestion with trypsin (Fig. 3C).^28^ The mixture of peptides obtained was analysed on a liquid chromatography-coupled trapped ion mobility-time of flight (timsTOF) mass spectrometer using data-dependant acquisition and label-free quantification. While diazirine probes have been reported to show differential affinity towards various amino acids,^29^ and have several reported off-targets,^30^ these were controlled for through competition experiments. Proteins were selected as hits if they were enriched by at least 2-fold in the probe-treated condition compared to treatment with probe plus a 10-fold excess of Navtemadlin (off-compete) and had a student's paired *t*-test (permutation-based FDR = 0.05, *S*_0_ = 0.1) *p*-value of less than 0.01. These were then filtered further by significant enrichment (*p* ≤ 0.01) in the probe-treated condition compared with the negative control (treatment with Navtemadlin), and not significantly (*p* ≤ 0.01) enriched in the off-compete condition, compared to the negative control (Fig. 3D and S7†). Of the remaining hits, proteins that were upregulated in the whole proteome of the probe-treated condition compared to the off-compete condition were also excluded. Both probes were used in these experiments, and each was tested in SJSA-1 and MCF-7 cell lines. The list of significant hits obtained is summarised in Fig. 3E. The only protein identified as a significant hit in more than one experiment was MDM2, appearing in three out of the four experiments. In the experiment conducted using probe 1 in the MCF-7 cell line, MDM2 was not detected. MDM2 is a low abundance protein, and has not previously been identified in photoaffinity labelling-based chemical proteomics experiments conducted with MDM2 inhibitors.^31^ A label-free proteome integral solubility alteration (PISA) experiment conducted by Van Vranken and coworkers using idasanutlin, a well-studied and potent inhibitor of MDM2, also failed to identify MDM2 by mass spectrometry.^32^ Therefore, the consistent appearance of this protein across multiple experiments gave confidence in the robust selectivity and affinity of Navtemadlin for its target protein. Compared to idasanutlin, which was also assessed for its selectivity by Zhu and coworkers through an ABPP approach, Navtemadlin showed a comparable degree of selectivity, with a similar number of off-targets being identified in a single experiment.^31^

Different off-targets were identified in the two cell lines which could not be attributed solely to differences in protein abundance (Fig. S2A†). Several cell membrane proteins were identified as significant hits with probe 1, but not with probe 2: gamma-glutamyltransferase 7 (GGT7), extended synaptotagmin 2 (ESYT2), and radixin (RDX).^33^ Meanwhile, off-target hits obtained using probe 2 were intracellularly located, in the cytoplasm, Golgi apparatus, endoplasmic reticulum and nucleus.^33^ Differences in the hits identified using the two probes reflect the significance of small changes in photoaffinity tag design in modulating cellular localisation and protein binding tendencies. This demonstrates that the use of a range of different minimalist tags can broaden the set of targets that can be identified, while enabling the selection of high-confidence hits that are minimally affected by subtle changes in probe design. The consistent identification of MDM2 across different probes and cell lines differentiates it as the only target that can be robustly confirmed. It is crucial to note, however, that potential off-targets engaged by the carboxylic acid moiety in Navtemadlin may have been overlooked in this study, as a result of the position at which the alkyne–diazirine modifications were introduced.

To gain a deeper understanding of the activity of Navtemadlin and the pathways affected, changes in global protein expression were analysed by mass spectrometry, 1, 4, 10, and 24 hours after treatment with 50 nM Navtemadlin (Fig. 4 and S8†). In both cell lines, the onset of activity was observed at the 4 h timepoint, indicated by the upregulation of MDM2, p21, and p53 expression. Changes in protein expression were found to intensify over time, with more p53 downstream proteins being upregulated. In addition, at the 10 h and 24 h timepoints, several proteins were found to be downregulated in the SJSA-1 cell line. Pathway analysis indicated that this set of proteins is involved in chromatin organisation, chromatin modification, DNA metabolic processes, and epigenetic regulation of gene expression.^34^ Strikingly, no proteins were found to be significantly downregulated in the MCF-7 cell line, mirroring the differences in downstream activity between the two cell lines. p53 is well-reported to induce distinct responses depending on the degree of cellular stress experienced; the SJSA-1 cell line displays a response typical of strong p53 activation, while MCF-7 cells exhibit a response observed upon a lower level of p53 activation, such as in the presence of reversible DNA damage.^35^ We believe this is primarily due to the difference in endogenous MDM2 expression between the two cell lines (Fig. S2†), however it may also be impacted by the differences in the expression of protein phosphatase 1D (PPM1D) between the two cell lines. PPM1D is a negative regulator of p53 and can prevent p53-mediated cell death upon inhibition of the p53-MDM2 PPI.^36^ This protein is significantly overexpressed in the MCF-7 cell line (Fig. S2A†),^37^ and may contribute to the lack of cytotoxicity observed in this cell line.

**Fig. 4:**
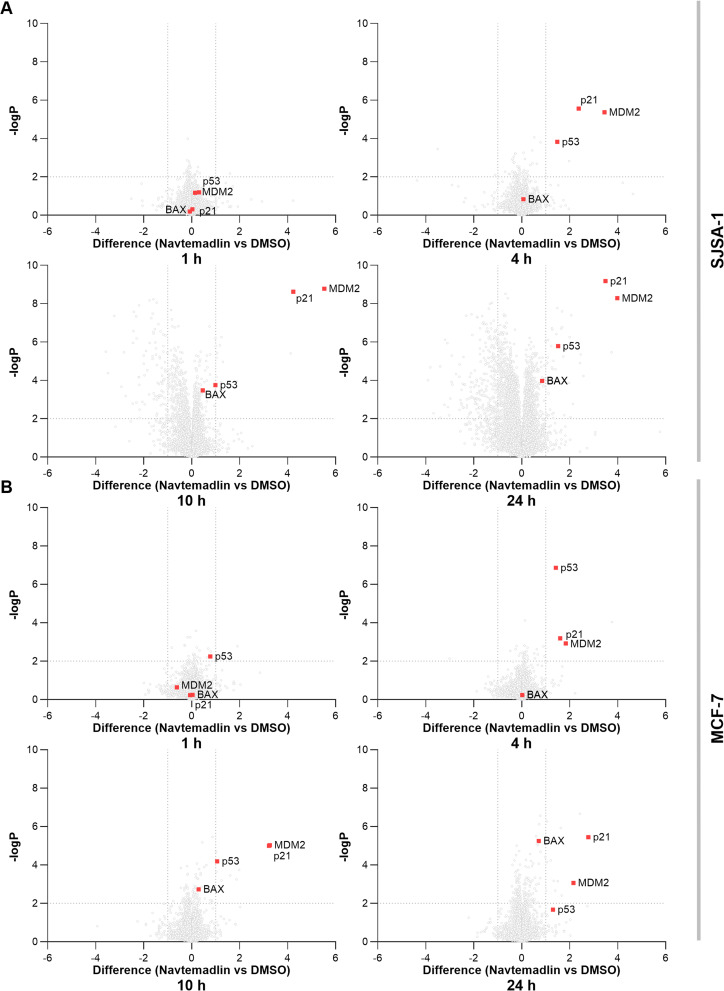
Time-course analysis of protein expression following treatment with 50 nM Navtemadlin. Differences in protein expression (*x*-axis) 1, 4, 10, and 24 h after treatment with Navtemadlin *versus* vehicle in SJSA-1 (A) and MCF-7 (B) cell lines. Associated significance (*y*-axis) is determined by paired Student's *t*-test (FDR = 0.05, *S*_0_ = 0.1, *n* = 4). Total of proteins quantified = 8747 in SJSA-1 and 8803 in MCF-7.

In summary, this unbiased target profiling approach, coupled with a time course analysis of the whole proteome following drug treatment, has confirmed MDM2 as the primary target of Navtemadlin. The development of selective PPI inhibitors has long been considered an ambitious feat, given the relatively large and shallow surfaces being targeted.^8^ These data reflect the exquisite selectivity of Navtemadlin for its target protein, despite these challenges. It also reaffirms the hypothesis that most adverse effects associated with the drug are likely to be a result of p53 activation in healthy cells, which could be overcome through effective dosing strategies.^17,38^

## Data availability

Raw data associated with this study is currently available through the Imperial College Data Repository: https://doi.org/10.14469/hpc/14879.

The mass spectrometry proteomics data have been deposited to the ProteomeXchange Consortium (https://www.proteomexchange.org) *via* the PRIDE partner repository.

Raw Data files can be found at https://doi.org/10.14469/hpc/14879 and the following DOIs:

**Table:** 

Data Type	DOI
NMR spectra	https://doi.org/10.14469/hpc/14880
Fluorescence anisotropy	https://doi.org/10.14469/hpc/14881
Cell toxicity (MTS, confluence, and cytotoxicity)	https://doi.org/10.14469/hpc/14882
Intact protein labelling	https://doi.org/10.14469/hpc/14883

The mass spectrometry proteomics data have been deposited to the ProteomeXchange Consortium (https://www.proteomexchange.org) *via* the PRIDE partner repository with the dataset identifiers listed below:^9^

**Table:** 

Experiment	Project accession code
Comparison of SJSA-1 and MCF-7 whole proteome after Navtemadlin treatment	PXD058081
Whole proteome experiment to validate treatment conditions in SJSA-1 cells	PXD058053
Whole proteome experiment to validate treatment conditions in MCF-7 cells	PXD058050
AfBPP of Navtemadlin probes in SJSA-1 cells	PXD058033
AfBPP of Navtemadlin probes in MCF-7 cells	PXD058036
Whole proteome time course experiment in SJSA-1 cells	PXD058053
Whole proteome time course experiment in MCF-7 cells	PXD058054

## Author contributions

A. B., E. W. T and A. J. W conceived and supervised the project. A. D, A. W, P. Z., J. W. H, J. L., A. T and T. K performed the experiments. All authors contributed to data analysis. A. D and A. B wrote the manuscript and all authors contributed to manuscript editing.

## Conflicts of interest

E. W. T is a founder and shareholder of Myricx Bio Ltd and Siftr Bio Ltd, an advisor to and holds share options in Samsara Therapeutics and Dunad Therapeutics, and receives current or recent funding from Myricx Bio Ltd, Pfizer Ltd, Kura Oncology, AstraZeneca, Merck & Co. and GSK. A. W is a founder and shareholder of Siftr Bio Ltd.

## Supplementary Material

SC-OLF-D5SC00120J-s001
