# Supplementary material for: Affinity-based protein profiling of MDM2 inhibitor Navtemadlin
Source: Chem Sci. 2025 Mar 12;16(16):6886–94. doi: 10.1039/d5sc00120j (PMC11921932; doi:10.1039/d5sc00120j)

Supplementary Information (SI) for Chemical Science.
This journal is © The Royal Society of Chemistry 2025

**Supplementary Information**

# Affinity-based protein profiling of MDM2 inhibitor Navtemadlin

Amrita Date[1], Archie Wall[1], Peiyu Zhang[2,3], Jack W. Houghton[1], Jianan Lu[1], Adam M. Thomas[1], Tristan Kovačič[1], Andrew J. Wilson[2,3,4], Edward W. Tate[1,5], Anna Barnard[1*]

1. Department of Chemistry, Molecular Sciences Research Hub, Imperial College London, 82 Wood Lane, London, W12 0BZ, United Kingdom
2. Astbury Centre for Structural Molecular Biology, University of Leeds, Woodhouse Lane, Leeds LS2 9JT, United Kingdom
3. School of Chemistry, University of Leeds, Woodhouse Lane, Leeds, LS2 9JT, United Kingdom
4. School of Chemistry, University of Birmingham, Edgbaston, Birmingham B15 2TT, United Kingdom
5. The Francis Crick Institute, London, NW1 1AT, United Kingdom

* Correspondence to: Dr Anna Barnard, Molecular Sciences Research Hub, Imperial College London, 82 Wood Lane, London W12 0BZ, United Kingdom. Email: a.barnard@imperial.ac.uk

## Contents

1. Supplementary Figures.......... 2
2. Experimental.......... 10
3. Online data repository.......... 18
3. References.......... 19
4. NMR spectra.......... 20
5. Uncropped blots.......... 22

## Supplementary Figures

| Compound | Bottom (anisotropy) | Top (anisotropy) | $IC_{50}$ (nM) | Hill Slope |
|---|---|---|---|---|
| **Navtemadlin** | -0.025 | 0.001 | 105 ± 12 | -2.04 |
| **Probe 1** | -0.026 | 0.002 | 361 ± 102 | -0.91 |
| **Probe 2** | -0.023 | 0.001 | 1005 ± 302 | -1.71 |

**Figure S1:** Curve parameters for fluorescence anisotropy assay.

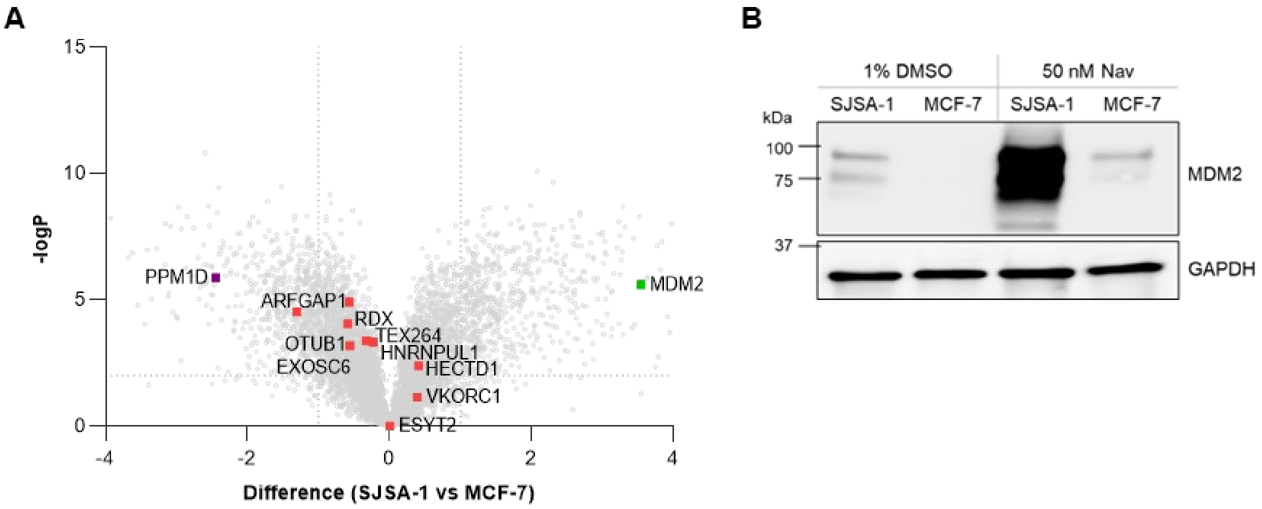


**Figure S2:** Differences in protein expression in SJSA-1 and MCF-7 cells. **(A)** Whole proteome analysis of SJSA-1 (right) and MCF-7 (left) cell lines treated with 50 nM Navtemadlin. Associated significance (y-axis) is determined by paired Student's t-test (FDR = 0.05, S0 = 0.1, n = 4). Proteins quantified = 6195. Proteins identified as statistically significant hits through AfBPP experiments shown in red, MDM2 in green, and PPM1D in purple. **(B)** Western blot showing differences in MDM2 expression between SJSA-1 and MCF-7 cells, treated with 50 nM Navtemadlin or vehicle.

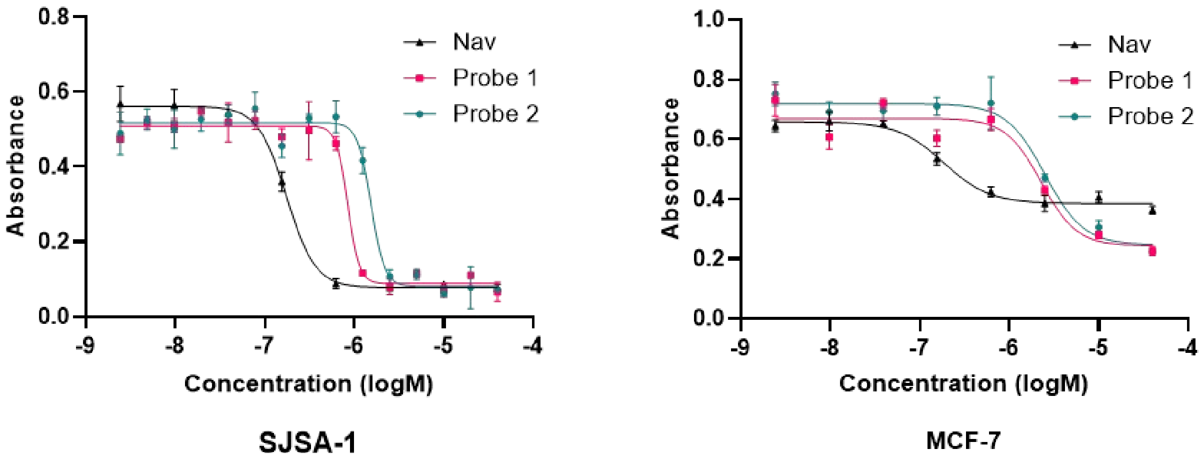


**Figure S3:** Quantification of in-cell anti-proliferative activity after 3 days of Navtemadlin/probe treatment through MTS assay (n=3) in SJSA-1 and MCF-7 cell lines. Resulting $EC_{50}$ values are summarised in Fig. 2B.

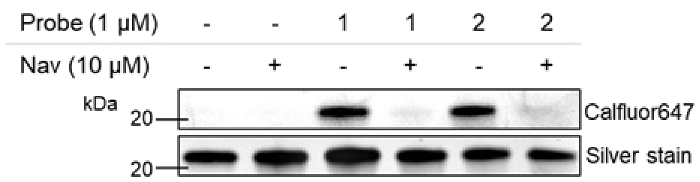


**Figure S4:** Validation of recombinant MDM2 labelling and availability of alkyne handle for click chemistry determined through in-gel fluorescence measurement following copper-catalysed click reaction with Calfluor-647 azide.

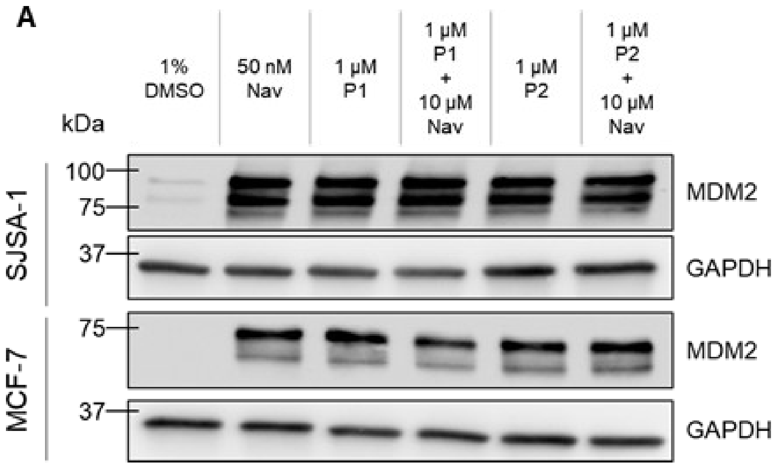
A
1% DMSO
50 nM Nav
1 µM P1
1 µM P1 + 10 µM Nav
1 µM P2
1 µM P2 + 10 µM Nav
kDa
100
75
37
SJSA-1
MCF-7
MDM2
GAPDH

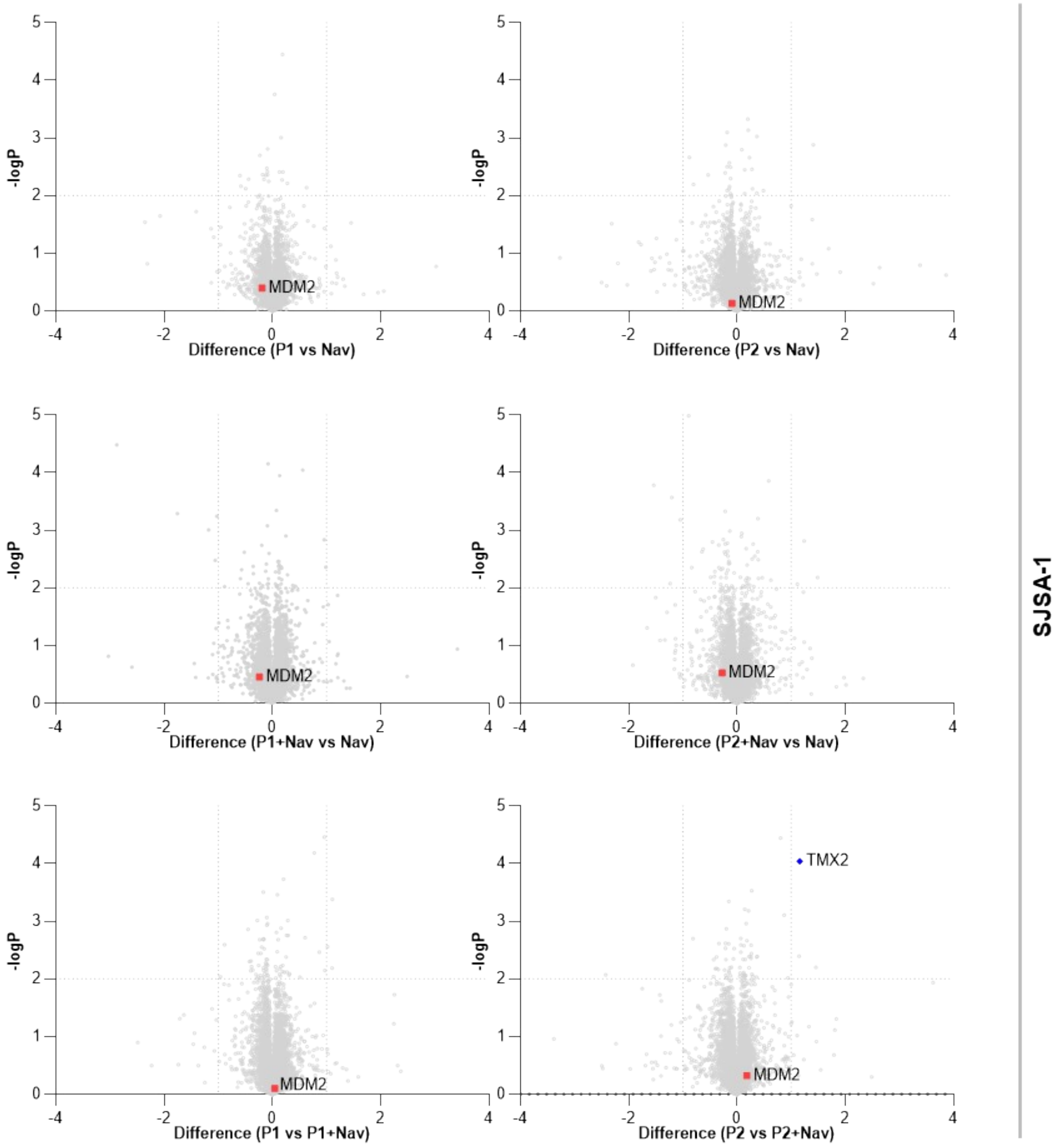
-logP
Difference (P1 vs Nav)
Difference (P2 vs Nav)
Difference (P1+Nav vs Nav)
Difference (P2+Nav vs Nav)
Difference (P1 vs P1+Nav)
Difference (P2 vs P2+Nav)
MDM2
TMX2
SJSA-1

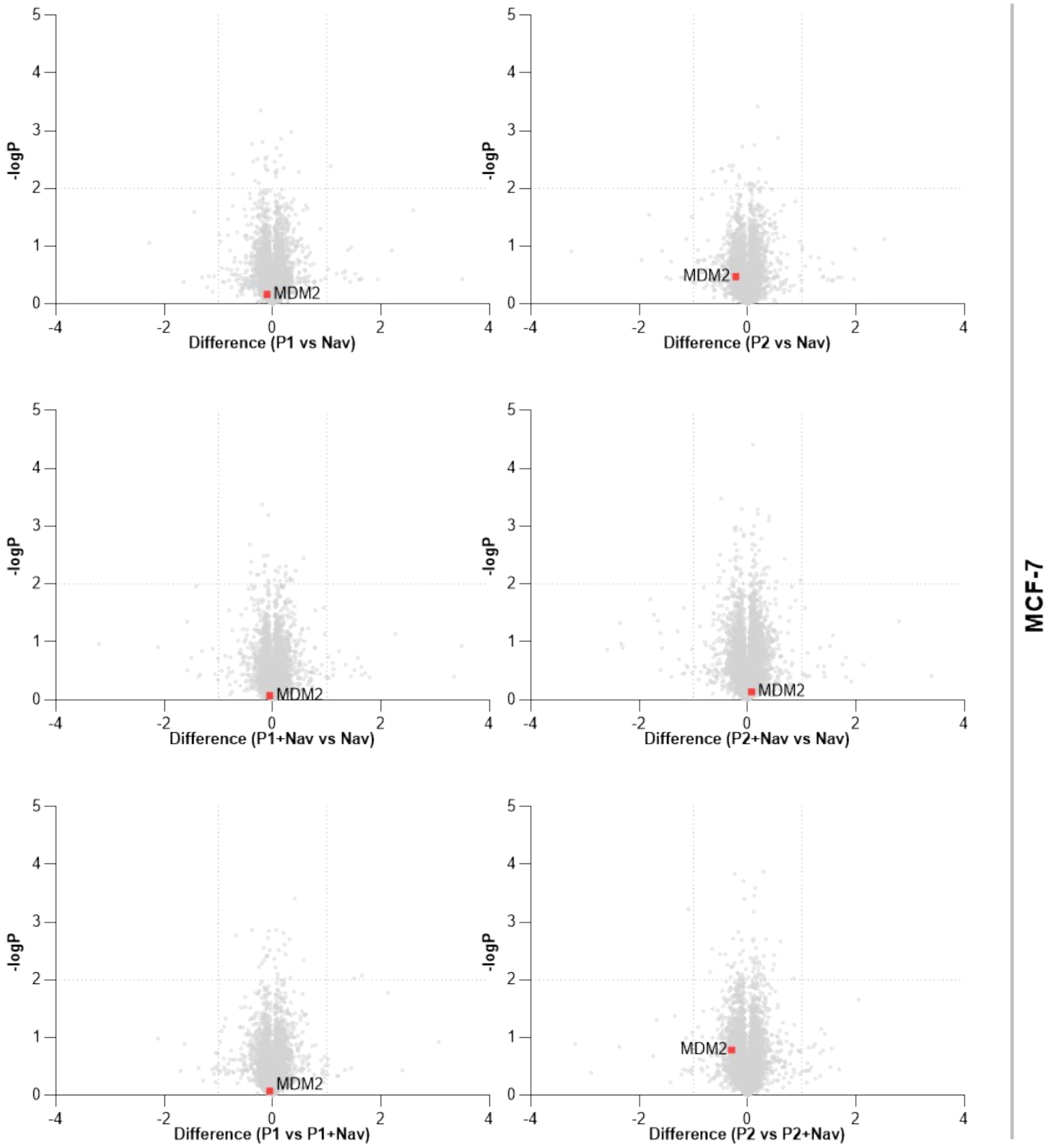


**Figure S5:** Validation of treatment conditions to ensure uniform expression of MDM2. **(A)** Western blot showing uniform MDM2 expression after treatment with 50 nM Navtemadlin or 1 μM probe or 1 μM probe + 10 μM Navtemadlin in SJSA-1 and MCF-7 cell lines. Whole proteome analysis of SJSA-1 **(B)** and MCF-7 **(C)** cell lines after treatment with 50 nM Navtemadlin or 1 μM probe or 1 μM probe + 10 μM Navtemadlin, to show that MDM2 expression is consistent across treatment conditions. Associated significance (y-axis) is determined by paired Student's t-test (FDR = 0.05, S0 = 0.1, n = 4 for SJSA-1, n = 3 for MCF-7). Proteins quantified = 9385 **(B)** and 8029 **(C)**.

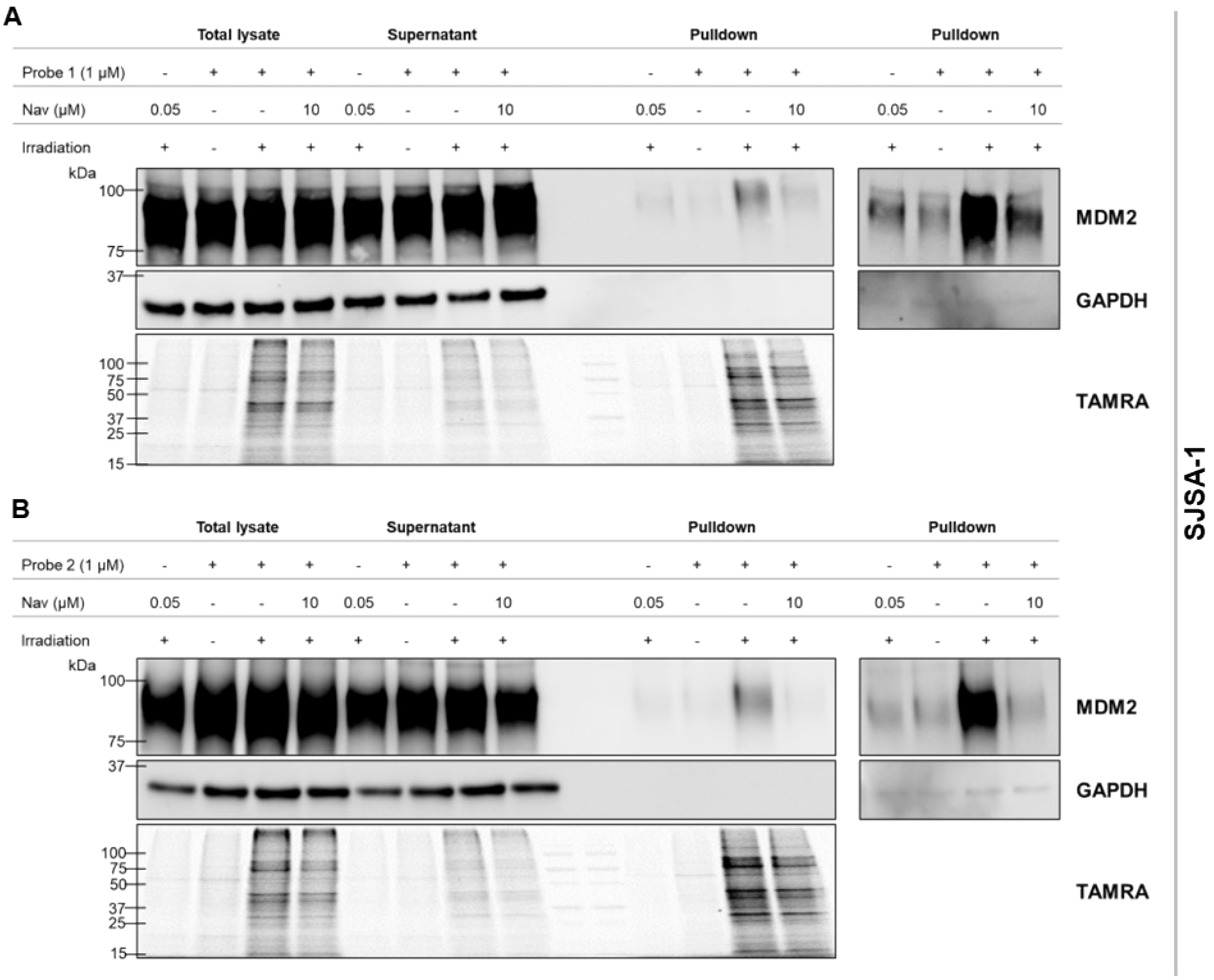
A
Total lysate
Supernatant
Pulldown
Pulldown
Probe 1 (1 µM)
Nav (µM)
Irradiation
kDa
MDM2
GAPDH
TAMRA
B
Total lysate
Supernatant
Pulldown
Pulldown
Probe 2 (1 µM)
Nav (µM)
Irradiation
kDa
MDM2
GAPDH
TAMRA
SJSA-1

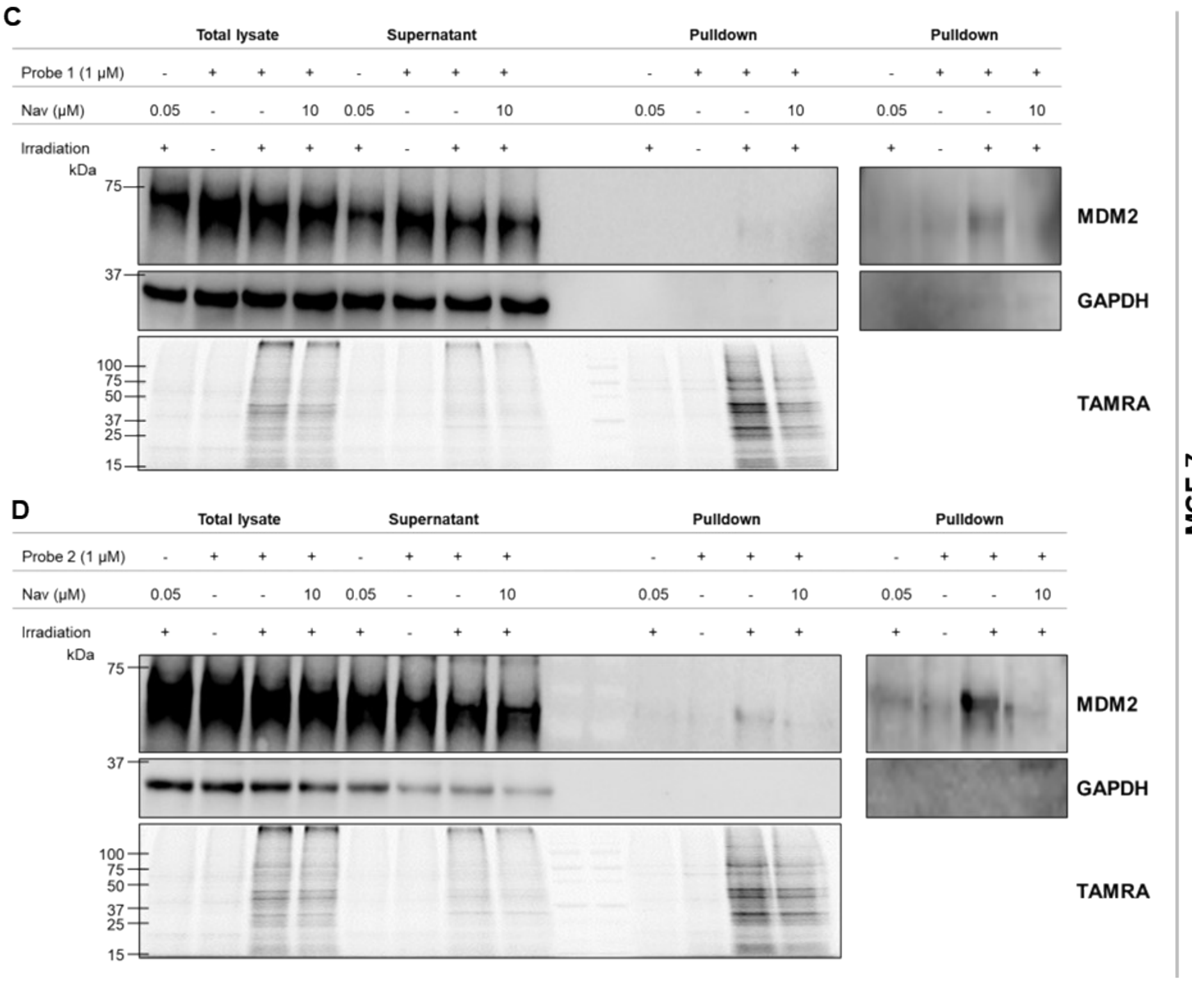


**Figure S6:** Western blot showing the light-dependent labelling and pulldown of MDM2 using photoaffinity probes in SJSA-1 (**A** and **B**) and MCF-7 (**C** and **D**) cell lines. Blots for MDM2 and GAPDH for total lysate, supernatant after enrichment on Neutravidin resin, and enriched protein (pulldown) have been imaged together. Blots for the enriched fraction were shown imaged separately at higher exposure to achieve better resolution. Overall protein labelling was detected as in-gel fluorescence for TAMRA.

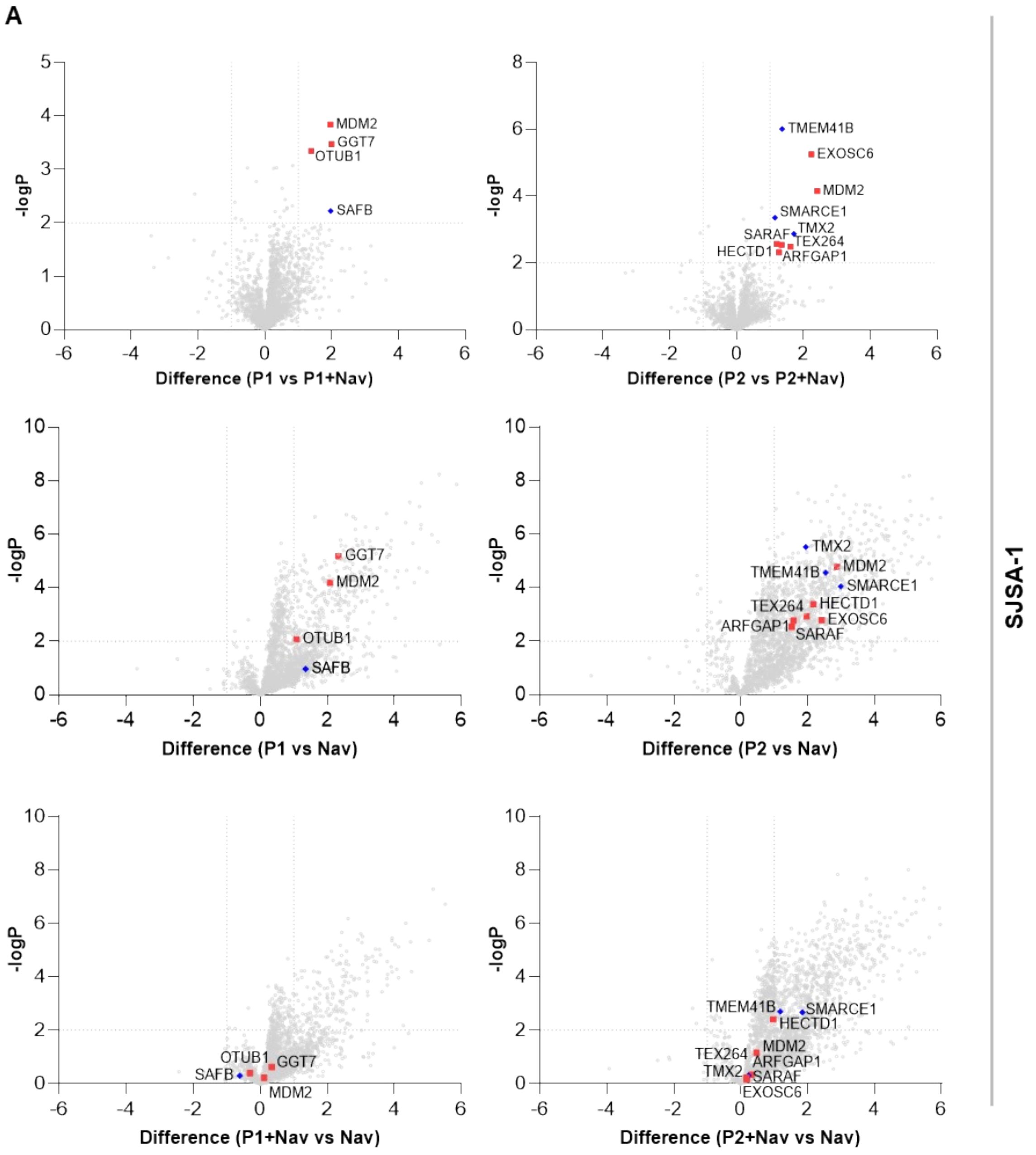
A
-logP
Difference (P1 vs P1+Nav)
MDM2
GGT7
OTUB1
SAFB
Difference (P2 vs P2+Nav)
TMEM41B
EXOSC6
MDM2
SMARCE1
SARAF
TMX2
TEX264
HECTD1
ARFGAP1
Difference (P1 vs Nav)
Difference (P2 vs Nav)
Difference (P1+Nav vs Nav)
Difference (P2+Nav vs Nav)
SJSA-1

B

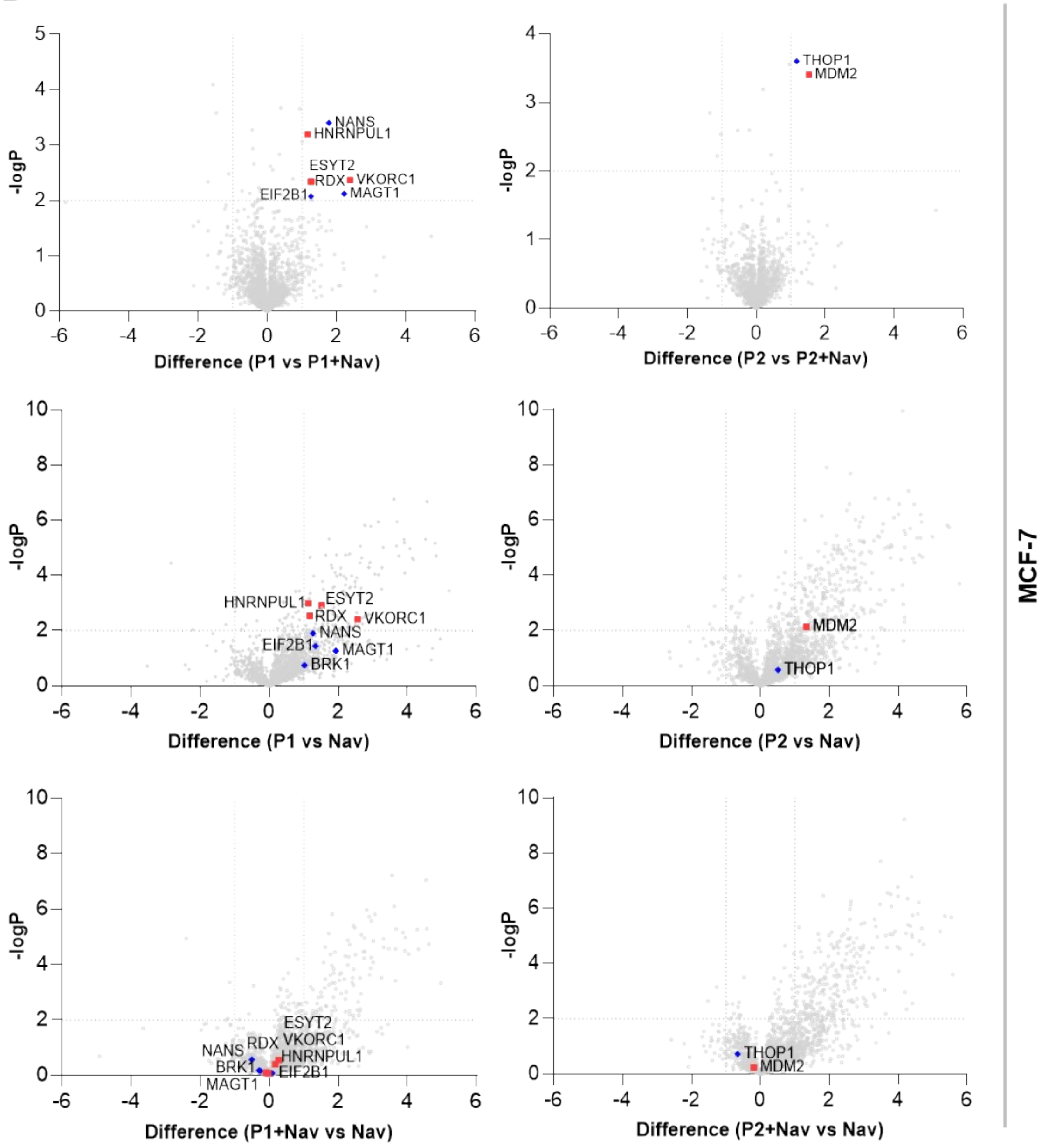


**Figure S7:** Target engagement profiles of Probe 1 and Probe 2 in SJSA-1 **(A)** and MCF-7 **(B)** cell lines. Volcano plots showing differences in enrichment (x-axis) between live cells treated with 1 µM probe ± 10 µM Navtemadlin (right) versus 50 nM Navtemadlin as a negative control (left) or 1 µM probe (right) versus 1 µM probe + 10 µM Navtemadlin (left). Associated significance (y-axis) is determined by paired Student's t-test (FDR = 0.05, S0 = 0.1, n = 4). Statistically significant hits identified are highlighted in red and hits that appear significant in volcano plots shown in the top row of each figure but have been disregarded due to insignificant labelling or off-compete, or due to differential expression in the whole proteome are shown in blue. Total number of proteins quantified = 2127 in SJSA-1 and 1797 in MCF-7.

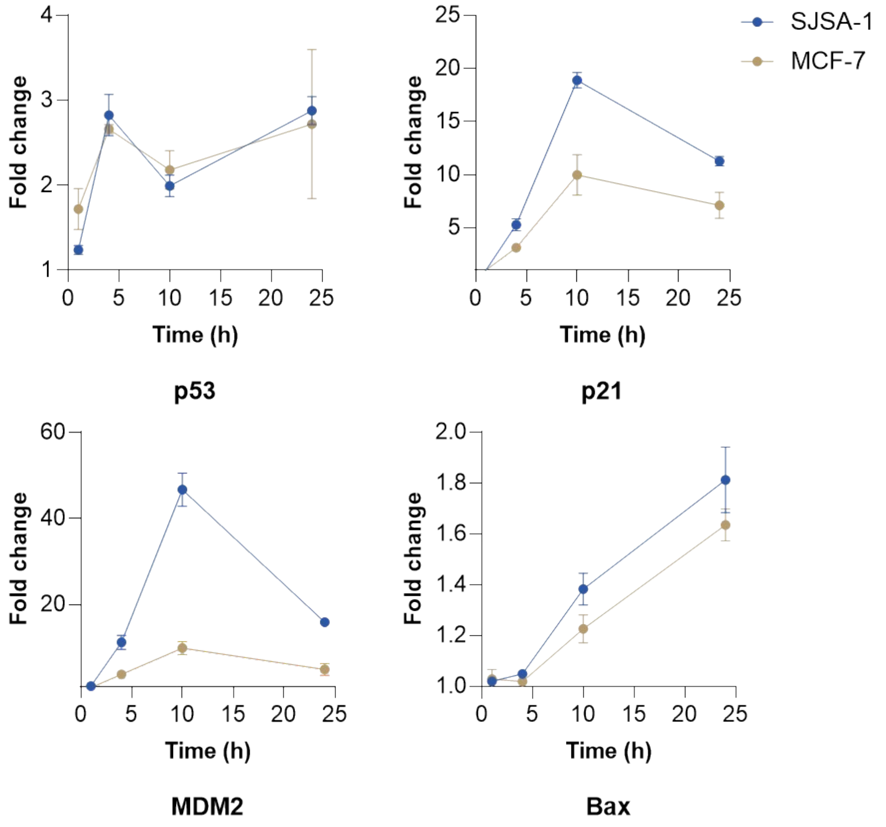


**Figure S8**: Time-course analysis of protein expression following treatment with 50 nM Navtemadlin. Changes in the expression of proteins of interest: p53, MDM2, p21, and Bax over time post Navtemadlin treatment (mean ± SEM, n=4).

## Synthesis

Reagents and solvents used in the synthesis and purification of the molecules were purchased as high-grade commercial products from Sigma-Aldrich, Fluorochem, VWR International or Alfa Aesar. Navtemadlin was purchased from MedChem Express. Azide-TAMTA-biotin (AzTB) was synthesised in-house as previously described.[1] N-(2-(3-methyl-3H-diazirin-3-yl)ethyl)prop-2-yn-1-aminium chloride was synthesised in-house as previously described.[2] Reactions were monitored by liquid chromatography-mass spectrometry (LC-MS), using a Micromass ZQ spectrometer, equipped with an XBridge C18 5 μm, 4.6 × 100 mm column. A gradient of 50–98% acetonitrile (MeCN) in water, both containing 0.1% formic acid, was run over 15 min at a flow rate of 1.2 mL/min. Purification of the final molecules was carried out by high performance liquid chromatography (HPLC), using a Shimadzu preparative HPLC system equipped with an Aeris Peptide 5 μm XB-C18 column. Nuclear magnetic resonance spectra were obtained using a Bruker Avance 400 MHz (1H, 400 MHz; 13C, 100 MHz) or a 500 MHz (1H, 500 MHz; 13C, 125 MHz) instrument. Chemical shifts (δ) have been reported in parts per million (ppm) and coupling constants (J) in hertz (Hz). Where applicable, splitting patterns have been described as singlet (s), doublet (d), triplet (t), quartet (q), and multiplet (m). The notation ‘i’ and ‘ii’ has been used to indicate non-equivalent protons attached to the same carbon.

**N-(2-(3-(but-3-yn-1-yl)-3H-diazirin-3-yl)ethyl)-2-((3R,5R,6S)-5-(3-chlorophenyl)-6-(4-chlorophenyl)-1-((S)-1-(isopropylsulfonyl)-3-methylbutan-2-yl)-3-methyl-2-oxopiperidin-3-yl)acetamide *(Probe 1)***

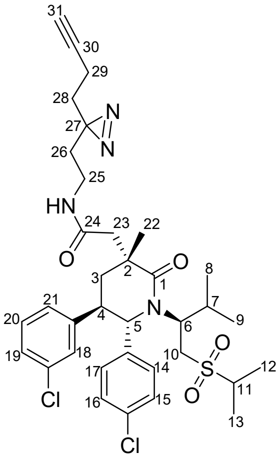

Synthesis was performed as described by Zhu *et al.*[3] To a solution of Navtemadlin (10 mg, 0.0176 mmol) in dimethylformamide (DMF; 0.2 mL), N,N-diisopropylethylamine (DIPEA; 6.92 µL, 0.0405 mmol, 2.3 eq.) and Hexafluorophosphate azabenzotriazole tetramethyl uronium (HATU, 10.02 mg, 0.0264 mmol, 1.5 eq.) were added. After 15 minutes, 2-(3-(but-3-yn-1-yl)-3H-diazirin-3-yl)ethan-1-amine (2.26 µL, 0.0176 mmol, 1 eq.) was added to the reaction mixture. The mixture was stirred in the dark. After 20 h, when complete conversion to the acid was observed by LC-MS, the reaction was quenched by the addition of water (2 mL). The product was extracted into ethyl acetate (3 x 10 mL). The organic phases were combined, washed with brine (3 x 10 mL), and dried over $MgSO_4$. Solvent was removed under reduced pressure, and the residue was purified by reverse-phase HPLC ($CH_3CN$:$H_2O$, 50–98%) to afford **Probe 1** as a white solid (6.6 mg, 55%). $^1$H NMR (500 MHz, MeOD) δ = 8.18 (bs, 3H, $H_{Ar}$), 7.18 (m, 5H, $H_{Ar}$), 5.10 (d, *J* = 11.0 Hz, 1H, $H_5$), 4.00 (dd, *J* = 13.9, 10.4 Hz, 1H, $H_{10i}$), 3.59 – 3.50 (m, 1H, $H_4$), 3.34 – 3.25 (m, 2H, $H_6$, $H_{11}$), 3.09 (tdd, *J* = 20.9, 13.9, 7.2 Hz, 3H, $H_{10ii}$, $H_{25}$), 2.88 (d, *J* = 13.5 Hz, 1H, $H_{23i}$), 2.48 (d, *J* = 13.5 Hz, 1H, $H_{23ii}$), 2.31 – 2.23 (m, 2H, $H_{3i}$, $H_{31}$), 2.18 (m, 1H, $H_7$), 2.08 (dd, *J* = 13.5, 3.1 Hz, 1H, $H_{3ii}$), 2.01 (td, *J* = 7.4, 2.7 Hz, 2H, $H_{29}$), 1.67 (ddd, *J* = 7.6, 6.9, 3.8 Hz, 2H, $H_{26}$), 1.62 (t, *J* = 7.4 Hz, 2H, $H_{28}$), 1.41 (d, *J* = 6.9 Hz, 6H, $H_{12}$, $H_{13}$), 1.35 (s, 3H, $H_{22}$), 0.66 (d, *J* = 6.6 Hz, 3H, $H_8$), 0.52 (d, *J* = 6.9 Hz, 3H, $H_9$). $^{13}$C NMR (126 MHz, MeOD) δ = 178.0, 172.7, 145.4, 139.0, 135.3, 135.2, 131.0, 129.3, 129.1, 128.0, 127.3, 83.6, 71.0, 70.4, 60.1, 55.7, 48.0, 45.5, 43.2, 40.4, 40.0, 35.4, 34.0, 33.5, 33.3, 27.8, 26.9, 21.2, 21.0, 16.0, 15.0, 13.8. LC-MS (50–98% MeCN) Rt = 11.60 min; m/z 687.7 ($[M+H]^+$). HRMS (m/z) calcd. for $[M+Na]^+$ $C_{35}H_{44}N_4O_4SCl_2$: 709.2358; found: 709.2348.

**2-((3R,5R,6S)-5-(3-chlorophenyl)-6-(4-chlorophenyl)-1-((S)-1-(isopropylsulfonyl)-3-methylbutan-2-yl)-3-methyl-2-oxopiperidin-3-yl)-N-(2-(3-methyl-3H-diazirin-3-yl)ethyl)-N-(prop-2-yn-1-yl)acetamide *(Probe 2)***

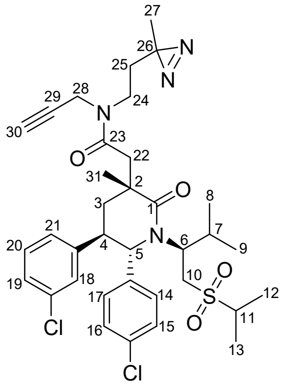

To a solution of Navtemadlin (10 mg, 0.0176 mmol) in DMF (0.2 mL), HATU (10.02 mg, 0.0264 mmol, 1.5 eq.) and DIPEA (7.04 µL, 0.0407 mmol, 2.3 eq.) were added. After 15 minutes, a solution of N-(2-(3-methyl-3H-diazirin-3-yl)ethyl)prop-2-yn-1-aminium chloride (3.08 mg, 0.0176 mmol, 1 eq.) and DIPEA (3.52 µL, 0.0209 mmol, 1.2 eq.) in DMF (0.1 mL) was added to the reaction mixture. The mixture was stirred in the dark for 20 h, until close to complete conversion to the amide was observed by LC-MS. The reaction was quenched by the addition of water (2 mL), and the product was extracted into ethyl acetate (3 x 10 mL). The organic phases were combined, washed with brine (3 x 10 mL), and dried over $MgSO_4$. Solvent was removed under reduced pressure. The crude product was purified by HPLC (MeCN:$H_2O$, 50–98%) to yield **Probe 2** as a white solid (5.4 mg, 45%). $^1$H NMR (400 MHz, MeOD) δ = 7.72 (bs, 3H, $H_{Ar}$), 7.16 (m, 5H, $H_{Ar}$), 5.09 (d, *J* = 10.7 Hz, 1H, $H_5$), 4.49 – 4.10 (m, 2H, $H_{28}$), 3.97 (dd, *J* = 13.8, 10.5 Hz, 1H, $H_{10i}$), 3.69 – 3.43 (m, 3H, $H_4$, $H_{24}$), 3.32 – 3.29 (m, 2H, $H_6$, $H_{11}$), 3.14 – 2.65 (m, 4H, $H_{10ii}$, $H_{22}$, $H_{30}$), 2.28 – 2.13 (m, 3H, $H_3$, $H_7$), 1.80 – 1.71 (m, 1H, $H_{25i}$), 1.66 (ddd, *J* = 8.3, 6.8, 4.5 Hz, 1H, $H_{25ii}$), 1.41 (d, *J* = 6.9 Hz, 6H, $H_{12}$, $H_{13}$), 1.37 (d, *J* = 9.6 Hz, 3H, $H_{31}$), 1.08 (d, *J* = 5.4 Hz, 3H, $H_{27}$), 0.68 (dd, *J* = 6.7, 4.2 Hz, 3H, $H_8$), 0.54 (dd, *J* = 7.0, 2.7 Hz, 3H, $H_9$). $^{13}$C NMR (101 MHz, MeOD) δ = 177.8, 145.5, 135.3, 135.2, 133.2, 130.8, 129.3, 129.2, 127.9, 127.4, 71.0, 60.2, 55.8, 45.8, 43.6, 43.0, 42.4, 42.3, 41.3, 40.9, 39.3, 34.3, 33.7, 30.7, 27.8, 21.2, 21.0, 19.7, 16.0, 15.1. LC-MS (50–98% MeCN) Rt = 13.30 min; m/z 687.7 ($[M+H]^+$). HRMS (m/z) calcd for $[M+H]^+$ $C_{35}H_{44}N_4O_4SCl_2$: 687.2539; found: 687.2539.

## Docking

Docking was carried out using Molecular Operating Environment (Chemical Computing Group, version 2022) using a ligand-bound co-crystal structure of MDM2 (PBD: 4JRG). The protein and the ligand were subjected to energy minimization using the QuickPrep function; default settings were used. The binding site of the existing ligand was defined as the binding pocket. Triangle matcher placement algorithm and rigid receptor refinement were applied. The poses were scored using the London dG and GBVI/WSA dG methods. A total of 30 poses were attempted and those with the top 5 scores were further analysed. The top-scoring pose has been presented.

## Fluorescence anisotropy

$hDM2_{17\text{-}135}$L33E was expressed and purified as previously described.[4, 5] A fluorescein-labelled p53 peptide tracer (Ac-SQETFSDLWKLLPENNVC$^{Flu}$-NH2, Flu-p53) was purchased from Peptide Protein

Research Ltd. A protein titration was carried out to determine the $K_d$ of this tracer, which was found to be 74.3 ± 4.2 nM.

The fluorescence anisotropy competition assay was carried out as previously described.[5, 6] Analyte solutions were prepared as 5 mM stocks in dimethylsulfoxide (DMSO) and diluted to 1 mM in 20 mM Tris, 150 mM NaCl, pH 7.6, 0.01% Triton-X-100. Serial dilutions of the analyte solutions were carried out in black flat-bottom 384-well plates (Grenier Bio-one) in triplicate. To this hDM2 and Flu-p53 solutions were added to a final concentration of 150 nM and 25 nM respectively. The highest working concentration of the analytes was 250 µM. The plate was read using an EnVisionTM 2103 MultiLabel plate reader (Perkin Elmer), using an excitation wavelength of 480 nm (30 nm bandwidth) and an emission wavelength of 535 nm (30 nm bandwidth). Intensity and anisotropy were calculated using the equations below:

$$I = (2PG) + S \qquad (1)$$

$$r = (S - PG)/I \qquad (2)$$

Where I = total intensity; P = perpendicular intensity; S = parallel intensity; G = instrument factor which was set to 1.1 for all assays; r = anisotropy. The data was fit to a [Inhibitor] vs. response – variable slope (four parameter) model using GraphPad Prism 10 (Dotmatics).

## Intact protein mass spectrometry

Recombinant $hDM2_{17\text{-}135}$L33E (2 µM) was treated with 4 µM probe ± 40 µM Navtemadlin in 1% DMSO in 4-(2-hydroxyethyl)-1-piperazineethanesulfonic acid buffer (HEPES, Sigma-Aldrich) pH 8.0. Samples treated with 1% DMSO and with 40 µM Navtemadlin were used as negative controls. Samples were agitated for 30 min at room temperature (rt), then irradiated at 365 nm for 10 min on ice. The experiment was carried out in triplicate, in a clear flat-bottom 96-well plate (Grenier). Liquid chromatography-mass spectrometry (LC-MS) analysis was carried out using an AdvanceBio 6545XT LC/Q-TOF system (Agilent) fitted with a ACQUITY UPLC Protein BEH C4 VanGuard Pre-column (Waters). Data was processed using BioConfirm (Agilent). The total ion chromatograms were extracted (region containing protein) and deconvoluted using MaxEnt. Percentage protein labelling was calculated using equation 3:

$$Percentage\ protein\ labelling = \frac{Labelled\ protein\ intensity}{Unlabelled\ protein\ intensity + Labelled\ protein\ intensity} \times 100 \qquad (3)$$

## Recombinant protein labelling gel

Aliquots of samples from the intact protein mass spectrometry experiment (20 µL) were subjected to a click reaction with CalFluor 647 azide (BroadPharm). A click master mix was prepared by mixing stock solutions of $CuSO_4$ (50 mM in water), sodium ascorbate (50 mM in water), and Tris(benzyltriazolylmethyl)amine (TBTA, 10 mM in DMSO) and CalFluor 647 azide (10 mM in DMSO) in a ratio of 2:2:1:1. To each sample, 1.5 µL of the click master mix was added and samples were agitated for 1 h at rt. Reaction was quenched by addition of ethylenediaminetetraacetic acid (EDTA, Sigma-Aldrich) to a final concentration of 10 mM. Colourless Laemmli loading buffer containing 5% 2-mercaptoethanol was added, samples were heated to 95 °C for 5 min, and protein was resolved by sodium dodecyl sulphate-polyacrylamide gel electrophoresis (SDS-PAGE). A Mini-PROTEAN TGX precast protein gel (4–15%, Bio-Rad) was used. The gel was imaged using the Typhoon™ FLA 9500 biomolecular imager, to detect the in-gel fluorescence.

The gel was stained using a Pierce™ silver stain kit (Thermo Scientific), as per the manufacturer's instructions.

## Cell culture

SJSA-1 cells were obtained from the Francis Crick Institute Cell Services facility. MCF-7 cells were obtained from CRUK Cell Services core facility. MCF-7 and SJSA-1 cells were cultured in low-glucose Dulbecco's Modified Eagle Medium (Sigma-Aldrich or Gibco) and Roswell Park Memorial Institute Medium 1640 (Gibco), respectively, each supplemented with 10% foetal bovine serum. Both cell lines were cultured at 37 °C in a 5% $CO_2$ humidified incubator.

## Cell proliferation and cytotoxicity assay

Cells were seeded in a clear, flat-bottom tissue culture-treated 48-well plate (Falcon) at a density of 5000 cells/well. After 24 h, once cells had adhered, they were treated in triplicate with the analytes in 0.5% DMSO. SYTOX green nucleic acid stain (Invitrogen) was added to a final concentration of 250 nM. Puromycin (2 µg/mL) was used as a positive control and 0.5% DMSO was used as a negative control. The plate was placed in an Incucyte S3 cell imager (Sartorius, Essen Bioscience). Green filter and phase readings were taken (4 images per well), every 4 h, over the course of 5 days. Data analysis was conducted using the integrated software (IncuCyte 2021B). The experiment was carried out as technical replicates (n=3). The mean ± standard error for the confluence and total green object integrated intensity per well/confluence per well (measured in green calibrated units, GCU) was plotted as a function of time using GraphPad Prism 10.

MTS assay was carried out using CellTiter 96® AQueous One Solution cell proliferation assay reagent (Promega), as per the manufacturer's instructions. Cells were seeded in a clear flat-bottom tissue culture-treated 96-well plate (Corning) at a density of 2000 cells/well. After 24 h, cells were treated with a range of probe concentrations in 1% DMSO in serum-containing media for 72 hours. Media was then replaced, the assay reagent was added, and the plate was incubated at 37 °C for 2.5–3 h. Absorbance was recorded at 480 nm using an Envision 2104 multilabel reader (PerkinElmer). The experiment was carried out as technical replicates (n=3). The data was fit to a [Inhibitor] vs. response – variable slope (four parameter) model using GraphPad Prism 10

## Western blot

Cells were observed under a microscope to ensure that they were healthy and at approximately 80–90% confluence. Media was aspirated and replaced with serum-free media containing molecules in 1% DMSO. Cells were incubated in the dark with the compounds for 4 h. The media was removed, and the cells were washed twice with phosphate buffered saline (PBS, Sigma-Aldrich). Cells were lysed on the plate using either i) radioimmunoprecipitation assay (RIPA) buffer (ThermoScientific) supplemented with complete protease inhibitor cocktail (Roche), or ii) with 1% sodium dodecyl sulphate (SDS, Sigma-Aldrich) in PBS supplemented with complete protease inhibitor cocktail (Roche) and Benzonase® nuclease (Millipore). A cell scraper was used to aid cell lysis. The protein content of the lysates was quantified using the DC protein assay (Bio-Rad), as per the manufacturer's instructions, and the protein concentration the samples was normalised to 1–2 mg/mL.

Proteins were resolved by SDS-PAGE. Mini-PROTEAN TGX precast protein gels (4–15%), were used. Laemmli loading buffer (Bio-Rad) with 5% 2-mercaptoethanol was added and the lysates were

heated to 95 °C for 5–10 min prior to loading. Proteins were blotted onto a 0.45 μm nitrocellulose blotting membrane (Amersham) using a Trans-Blot Turbo Transfer system (Bio-Rad), as per the manufacturer's instructions. The membrane was blocked using 5% milk in tris-buffered saline with 0.1% Tween 20 (TBST) for 1 h at rt. Primary antibodies for p21 (Cell Signalling Technology, mAb2947), MDM2 (Abcam, ab16895), and GAPDH (Abcam) were diluted (1:1000) in 5% milk in TBST. Blocked membranes were incubated with the primary antibodies for 1 h at rt. The membranes were then washed with TBST, before incubating with a horseradish peroxidase (HRP)-conjugated secondary antibody (Advansta) at rt for 1 h. Membranes were then washed (3 x 5 min) with TBST, stained with Immobilon Crescendo Western HRP substrate (Millipore), and imaged for chemiluminescence using an ImageQuant LAS-4000 imaging system (GE Healthcare).

**Whole proteome proteomics**

All sample preparation for proteomics was carried out in Protein LoBind microcentrifuge tubes (Eppendorf).

Cells were treated in serum-free media containing the molecules in 1% DMSO and incubated at 37 °C for 4 h. The media was removed, and the cells were washed with PBS (x2). Cells were lysed using 1% SDS in PBS supplemented with complete protease inhibitor cocktail and Benzonase® nuclease. The protein concentration of the lysates was quantified using the DC protein assay and normalised to 1-2 mg/mL. Reduction and alkylation of the proteins was carried out by treating with 10 mM tris(2-carboxyethyl)phosphine (TCEP, Sigma-Aldrich) and 40 mM chloroacetamide (CAA, Sigma-Aldrich) for 20 min at rt followed by 20 min at 37 °C. Protein was then precipitated by the addition of 4 volumes of acetonitrile (VWR, HPLC grade). The sample was centrifuged, and the supernatant discarded. The pellet was then washed with 80% ethanol (VWR, HPLC grade) (x3). The protein pellet was then treated with trypsin (6.25 ng/μL, Pierce) in a 25 mM ammonium bicarbonate (SigmaAldrich) pH 8.0 buffer and agitated at 37 °C overnight. The samples were checked for the disappearance of the protein pellet at the end of the incubation period and dried using a SpeedVac concentrator.

**Click and Pulldown**

The procedure was modified from that previously described.[2, 7, 8]

*Photo-crosslinking*

Cells were treated in serum-free media containing the probes in 1% DMSO and incubated in the dark for 4 h. The media was removed, and the cells were washed with PBS. PBS was added to cover the surface of the plate. The plates were placed on ice and irradiated with ultraviolet light (365 nm) for 120 s. The PBS was then removed, and cells were lysed using 1% SDS in PBS supplemented with complete protease inhibitor cocktail and Benzonase® nuclease. The DC protein assay (Bio-Rad) was used to determine the protein content of the lysates, and protein concentration was normalised to 1–2 mg/mL.

*Click reaction and protein precipitation*

A click master mix was prepared by mixing stock solutions of AzTB/ azide- PEG3-biotin (Sigma-Aldrich) (10 mM in DMSO), $CuSO_4$ (50 mM in water), sodium ascorbate (50 mM in water), and TBTA (10 mM in DMSO) in a ratio of 1:2:2:1. The mixture was vortexed and allowed to stand for 2 min

before adding 6 µL of the master mix per 100 µL of lysate. The reaction was left to shake at rt for 1 h, and then quenched by the addition of EDTA to a final concentration of 5 mM.

Proteins were then precipitated by addition of 1 volume of methanol and 0.25 volumes of chloroform to the lysate. The mixtures were vortexed and centrifuged at 4 °C, 17,000 x *g* for 5 min. The supernatant was discarded, and the pellet was washed with methanol (2.5 vol x 2). The protein pellet was allowed to air dry, before redissolving in 0.2% SDS in 50 mM HEPES pH 8.0 to a final protein concentration of approximately 1 mg/mL (50% protein loss during precipitation was assumed).

*Pulldown*

Biotin enrichment was carried out using Neutravidin agarose resin (ThermoScientific). The resin was washed with 0.2% in 50 mM HEPES pH 8.0 (x3). One volume of resin was added to every 2.5 volumes of lysate, and the samples were agitated for 2 h at rt. The samples were then centrifuged, and the supernatant was discarded. The resin was washed with 0.2% SDS in 50 mM HEPES pH 8.0 (x3).

To analyze by Western blot, the captured proteins were eluted from the resin by adding 2x Lamelli loading buffer with 5% 2-mercaptoethanol and heating to 95 °C for 10 min. The samples were centrifuged, and the supernatant was collected for analysis by SDS-PAGE and Western blot. The gels were imaged using the Typhoon™ FLA 9500 biomolecular imager, to detect the in-gel fluorescence from TAMRA. Western blot was carried out as previously described.

## Pulldown proteomics

For analysis by proteomics, lysate containing 800 µg of protein at 2 mg/mL was used. Cell treatment, photo-crosslinking, protein precipitation and pulldown was carried out as previously described.[2, 7, 8]

Following the three washes with 0.2% SDS in 50 mM HEPES pH 8.0, the resin was further washed with 50 mM HEPES pH 8.0 (x3). The beads were then resuspended in 50 mM HEPES pH 8.0 and treated with 10 mM TCEP (Bond-breaker™ ThermoScientific) and 40 mM CAA. The samples were agitated for 10 min at rt, after which they were centrifuged, and the supernatant was removed. The resin was washed with 50 mM HEPES pH 8.0 (x2) and resuspended in 100 µL 50 mM HEPES pH 8.0. To each sample, 0.2 µg of trypsin (Pierce) was added and samples were agitated at 37 °C overnight. The samples were centrifuged (17,000 x *g*, 5 min) and the supernatant was collected. The resin was washed with 50 µL of 50 mM HEPES pH 8.0, centrifuged and the supernatants were combined. The collected peptide mixture was acidified with formic acid to a pH below 3. Samples were filtered through 0.45 µm hydrophilic Durapore® filters (Millipore). These samples were frozen at -20 °C until they were ready for processing.

## LC-MS/MS analysis

Peptides for proteomics analysis were loaded onto Evotips (Evosep) as per the manufacturer's instructions. For whole proteome analysis, 200 ng peptide was loaded, while for pulldown sample, half of the sample collected was loaded for analysis.

*For whole proteome experiments:*

Peptides were analysed by nanoLC-MS/MS using an Evosep One (Evosep) coupled with a timsTOF HT (Bruker) equipped with an 8 cm × 150 µm, 1.5 µm analytical column (Evosep). 200 ng peptides were separated by the Evosep 60SPD workflow (Analytical solvents A: 0.1% FA and B: acetonitrile

plus 0.1% FA). For the experiment shown in Figure S2, a 15 cm × 150 µm, 1.5 µm analytical column (Evosep) was used and 200 ng peptides were separated using the Evosep 30SDP workflow. Column was held at 40 °C. Data were acquired in data-independent acquisition (DIA) PASEF mode with the following settings: m/z range from 100 m/z to 1700 m/z, ion mobility range from 1/K0 = 1.30 to 0.85 Vs/cm$^2$ using equal ion accumulation and ramp times in the dual TIMS analyser of 100 ms each. Each cycle consisted of 8 PASEF ramps covering 21 mass steps each with 25 Da windows each with 2/3 non-overlapping ion mobility windows covering the 475 to 1000 m/z range and 0.85 and 1.26 Vs/cm$^2$ ion mobility range. The collision energy was lowered as a function of increasing ion mobility from 59 eV at 1/K0 = 1.6 Vs/cm$^2$ to 20 eV at 1/K0 = 0.6 Vs/cm$^2$.

*For pulldown experiments:*

Peptides were analysed by nanoLC-MS/MS using an Evosep One (Evosep) coupled with a timsTOF HT (Bruker) equipped with an 8 cm × 150 µm, 1.5 µm analytical column (Evosep). 200 ng peptides were separated by the Evosep 60SPD workflow (Analytical solvents A: 0.1% FA and B: acetonitrile plus 0.1% FA). Column was held at 40 °C. Data were acquired in data-dependent acquisition (DDA) PASEF mode with the following settings: m/z range from 100 m/z to 1700 m/z, ion mobility range from 1/K0 = 1.30 to 0.85 Vs/cm$^2$ using equal ion accumulation and ramp times in the dual TIMS analyzer of 100 ms each. The collision energy was lowered as a function of increasing ion mobility from 59 eV at 1/K0 = 1.6 Vs/cm$^2$ to 20 eV at 1/K0 = 0.6 Vs/cm$^2$. Isolation width was lowered as a function of decreasing m/s from 3 m/z at 800 m/z to 2 m/z at 700 m/z. Active exclusion was applied for 0.4 min. Each cycle consistent of 4 PASEF ramps (total cycle time 0.53 s) with 2.75 ms measuring time allowed for each selected precursor.

### Data processing for DIA

diaPASEF Bruker .d files were processed using library-free analysis in DIA-NN (version 1.8.1) [9] using the following parameters: Human database (Downloaded from UniProt 13 July 2023 containing 20552 proteins and 246 common contaminants; for experiment shown in Figure S2, database downloaded from Uniprot on 6 June 2022 containing 79334 proteins); "deep learning-based spectra and RTs prediction" was enabled; trypsin with 1 missed cleavages; N-term Excision, C carbamidomethylation, Oxidation and N terminal Acetylation were enabled with maximum 2 variable modifications; MBR was enabled; quantification strategy set to "Robust LC (high precision)"; heuristic protein inference was disabled; Mass and MS1 accuracy set to 0.

### Data processing for DDA

ddaPASEF Bruker .d files were processed using Fragpipe version 20.0 (Nesvilab).[10] Data was searched against a human reference proteome with isoforms (Uniprot, UP000005640, accessed 6 June 2022, 203368 proteins) with 50% decoys and contaminants added. The built-in label-free quantification-match between runs workflow was used. Bruker .d files were searched using MSFragger (version 3.8) The following parameters were used: strict trypsin digestion; a maximum of 2 missed cleavages allowed; precursor ion tolerance of 20 ppm; trimming of protein N-terminal methionine; oxidation (M) and N terminal acetylation as variable modifications; carbamidomethylation (C) as a fixed modification. MaxLFQ minimum ions was set to 1 and the retention time tolerance for match between runs was set to 2 min. All other default parameters were used for processing. MSFragger search results were processed using Percolator (version 3.5) for peptide-spectrum match validation, followed by Philosopher (version 5.0.0) for protein and FDR

filtering. Label-free quantification values were calculated using the MaxLFQ algorithm using IonQuant (version 1.9.8) with match between runs enabled and min ions set as 1.

### Data analysis using Perseus

Data analysis was carried out using Perseus version 1.6.2.3. Intensities (DIA data) or MaxLFQ intensities (DDA data) were loaded for analysis. A text filter was applied to remove contaminants and data was log2 transformed. Rows were annotated to group replicates, and data was filtered for valid values in at least three replicates in at least one condition. Data was normalized by subtracting the median from the columns and missing values were imputed based on normal distribution. Volcano plots were generated by plotting the fold-changes of the protein intensities against the -logp values for their significance. These were calculated by carrying out a two-sample t-test of the intensity values for each protein in the two conditions (permutation-based FDR = 0.05, S0 = 0.1). Data was plotted in GraphPad Prism version 10 and x- and y-axis cut-offs were set to ±1 and 2 respectively.

## Online data repository

Raw Data files can be found at 10.14469/hpc/14879 and the following DOIs:

| Data Type | DOI |
|---|---|
| NMR Spectra | 10.14469/hpc/14880 |
| Fluorescence Anisotropy | 10.14469/hpc/14881 |
| Cell toxicity (MTS, confluence, and cytotoxicity) | 10.14469/hpc/14882 |
| Intact Protein Labelling | 10.14469/hpc/14883 |

The mass spectrometry proteomics data have been deposited to the ProteomeXchange Consortium (https://www.proteomexchange.org) via the PRIDE partner repository with the dataset identifiers listed below:[11]

| Experiment | Project accession code |
|---|---|
| Comparison of SJSA-1 and MCF-7 whole proteome after Navtemadlin treatment | PXD058081 |
| Whole proteome experiment to validate treatment conditions in SJSA-1 cells | PXD058053 |
| Whole proteome experiment to validate treatment conditions in MCF-7 cells | PXD058050 |
| AfBPP of Navtemadlin probes in SJSA-1 cells | PXD058033 |
| AfBPP of Navtemadlin probes in MCF-7 cells | PXD058036 |
| Whole proteome time course experiment in SJSA-1 cells | PXD058053 |
| Whole proteome time course experiment in MCF-7 cells | PXD058054 |

## References


1. M. Broncel, R. A. Serwa, P. Ciepla, E. Krause, M. J. Dallman, A. I. Magee and E. W. Tate, Multifunctional reagents for quantitative proteome-wide analysis of protein modification in human cells and dynamic profiling of protein lipidation during vertebrate development, *Angew Chem Int Ed Engl*, 2015, **54**, 5948-5951.
2. C. R. Kennedy, A. Goya Grocin, T. Kovacic, R. Singh, J. A. Ward, A. R. Shenoy and E. W. Tate, A Probe for NLRP3 Inflammasome Inhibitor MCC950 Identifies Carbonic Anhydrase 2 as a Novel Target, *ACS Chem Biol*, 2021, **16**, 982-990.
3. D. Zhu, H. Guo, Y. Chang, Y. Ni, L. Li, Z. M. Zhang, P. Hao, Y. Xu, K. Ding and Z. Li, Cell- and Tissue-Based Proteome Profiling and Dual Imaging of Apoptosis Markers with Probes Derived from Venetoclax and Idasanutlin, *Angew Chem Int Ed Engl*, 2018, **57**, 9284-9289.
4. A. I. Green, F. Hobor, C. P. Tinworth, S. Warriner, A. J. Wilson and A. Nelson, Activity-Directed Synthesis of Inhibitors of the p53/hDM2 Protein-Protein Interaction, *Chemistry*, 2020, **26**, 10682-10689.
5. J. P. Plante, T. Burnley, B. Malkova, M. E. Webb, S. L. Warriner, T. A. Edwards and A. J. Wilson, Oligobenzamide proteomimetic inhibitors of the p53-hDM2 protein-protein interaction, *Chem Commun (Camb)*, 2009, DOI: 10.1039/b908207g, 5091-5093.
6. L. Liu, B. He, J. A. Miles, W. Wang, Z. Mao, W. I. Che, J. Lu, X. Chen, A. J. Wilson, D. Ma and C. Leung, Inhibition of the p53/hDM2 protein-protein interaction by cyclometallated iridium(III) compounds, *Oncotarget*, 2016, **7**, 13965-13975.
7. W. W. Kallemeijn, T. Lanyon-Hogg, N. Panyain, A. Goya Grocin, P. Ciepla, J. Morales-Sanfrutos and E. W. Tate, Proteome-wide analysis of protein lipidation using chemical probes: in-gel fluorescence visualization, identification and quantification of N-myristoylation, N- and S-acylation, O-cholesterylation, S-farnesylation and S-geranylgeranylation, *Nat Protoc*, 2021, **16**, 5083-5122.
8. R. T. Howard, P. Hemsley, P. Petteruti, C. N. Saunders, J. A. Molina Bermejo, J. S. Scott, J. W. Johannes and E. W. Tate, Structure-Guided Design and In-Cell Target Profiling of a Cell-Active Target Engagement Probe for PARP Inhibitors, *ACS Chem Biol*, 2020, **15**, 325-333.
9. V. Demichev, C. B. Messner, S. I. Vernardis, K. S. Lilley and M. Ralser, DIA-NN: neural networks and interference correction enable deep proteome coverage in high throughput, *Nat Methods*, 2020, **17**, 41-44.
10. F. Yu, S. E. Haynes, G. C. Teo, D. M. Avtonomov, D. A. Polasky and A. I. Nesvizhskii, Fast Quantitative Analysis of timsTOF PASEF Data with MSFragger and IonQuant, *Mol Cell Proteomics*, 2020, **19**, 1575-1585.
11. Y. Perez-Riverol, J. W. Bai, C. Bandla, D. García-Seisdedos, S. Hewapathirana, S. Kamatchinathan, D. J. Kundu, A. Prakash, A. Frericks-Zipper, M. Eisenacher, M. Walzer, S. B. Wang, A. Brazma and J. A. Vizcaíno, The PRIDE database resources in 2022: a hub for mass spectrometry-based proteomics evidences, *Nucleic Acids Res*, 2022, **50**, D543-D552.

# NMR Spectra

## $^{1}$H NMR of **Probe 1** at rt in $CD_3OD$

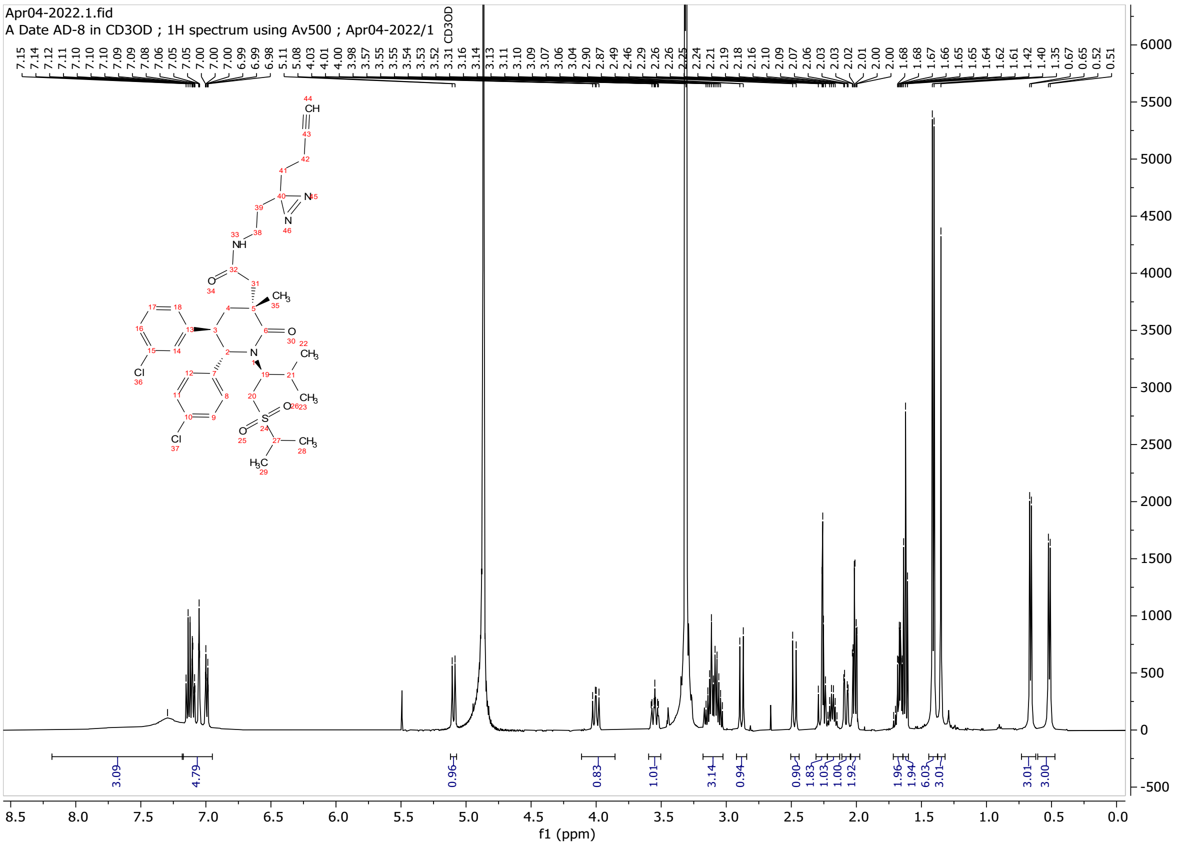


## $^{13}$C NMR of **Probe 1** at rt in $CD_3OD$

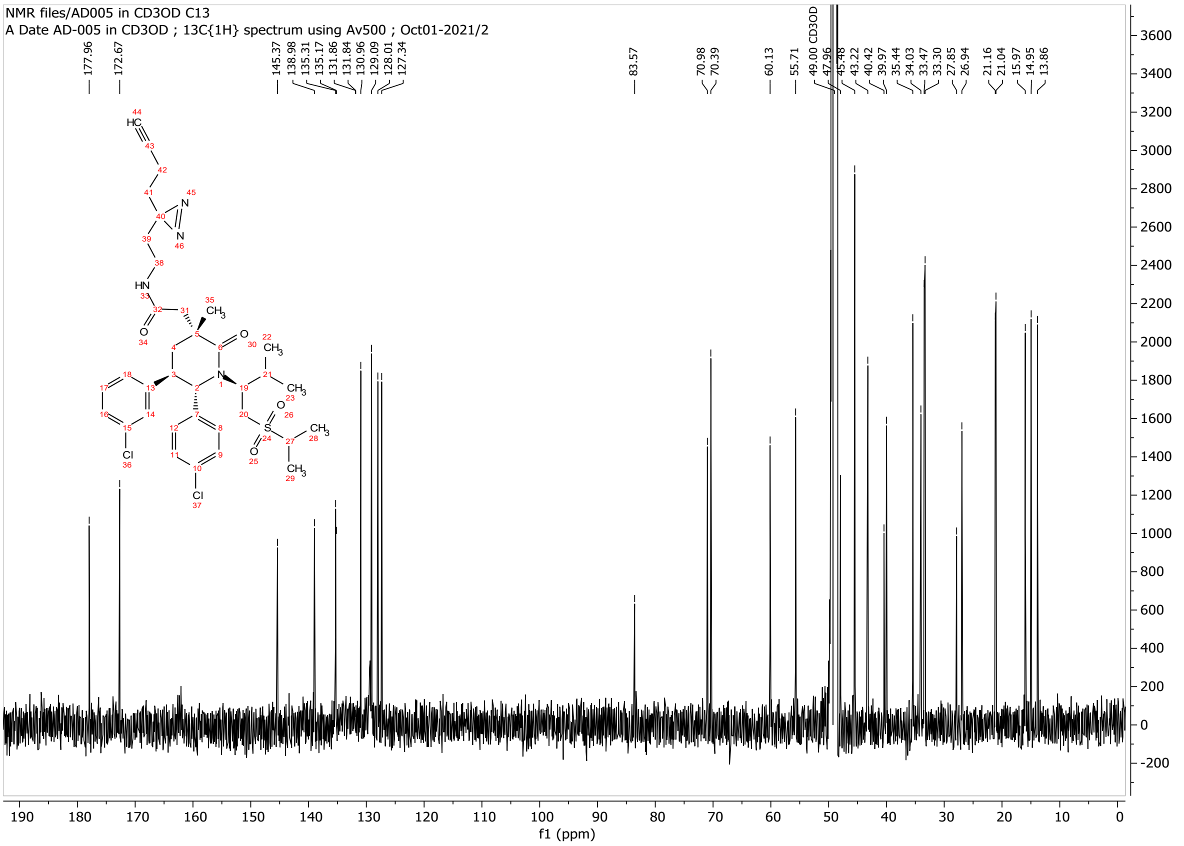

## $^1H$ NMR of **Probe 2** at rt in $CD_3OD$

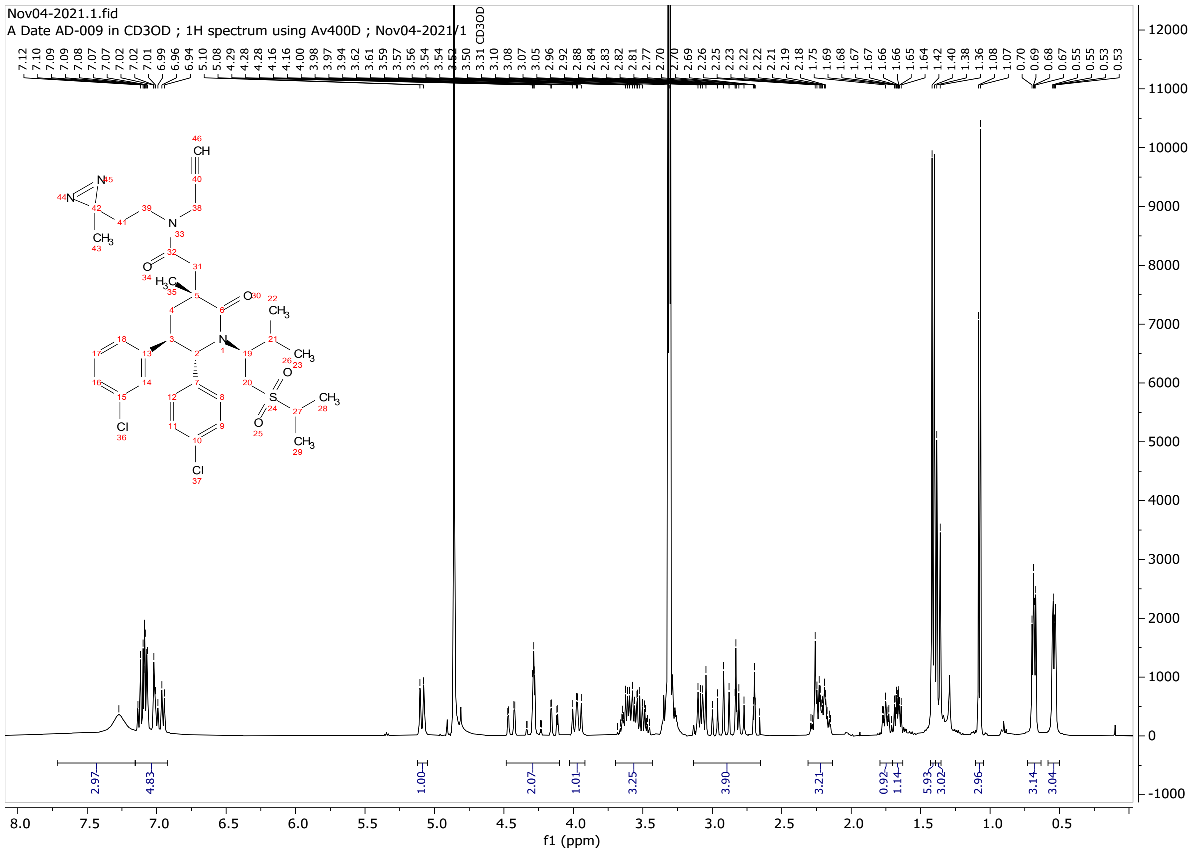


## $^{13}C$ NMR of **Probe 2** at 333 K in $CD_3OD$

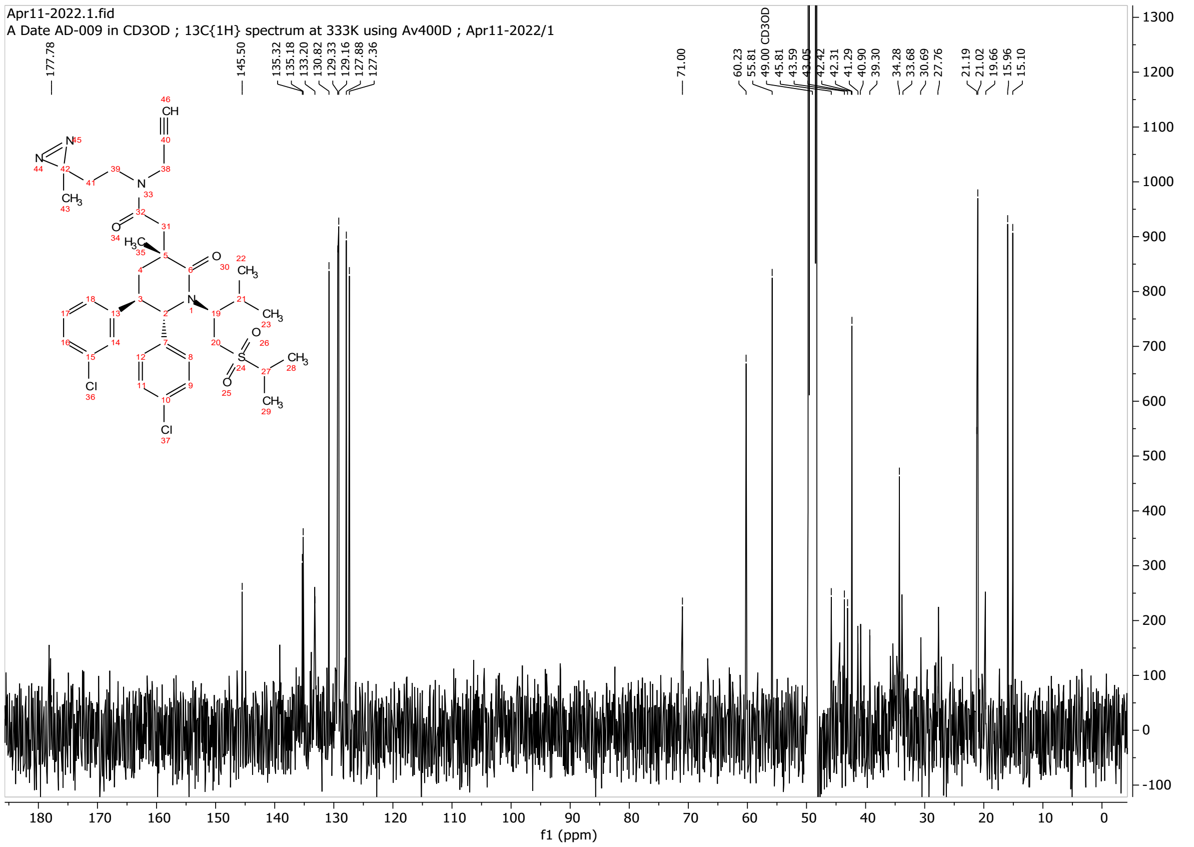

*COSY of **Probe 2** at rt in $CD_3OD$*

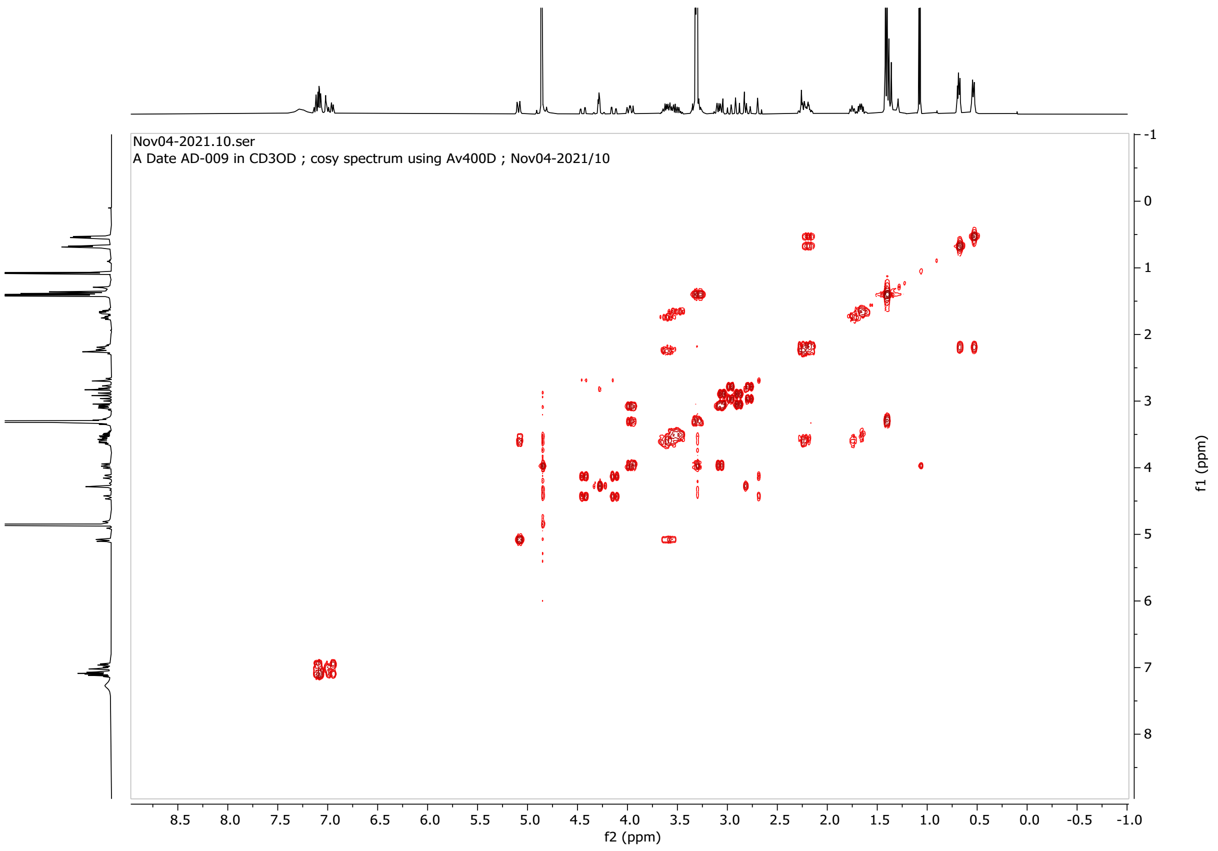


## Uncropped blots

Uncropped gels and Western blots. Where a portion of the blot has been cropped and shown, this is indicated by a red box around the region used.

### Figure 2B

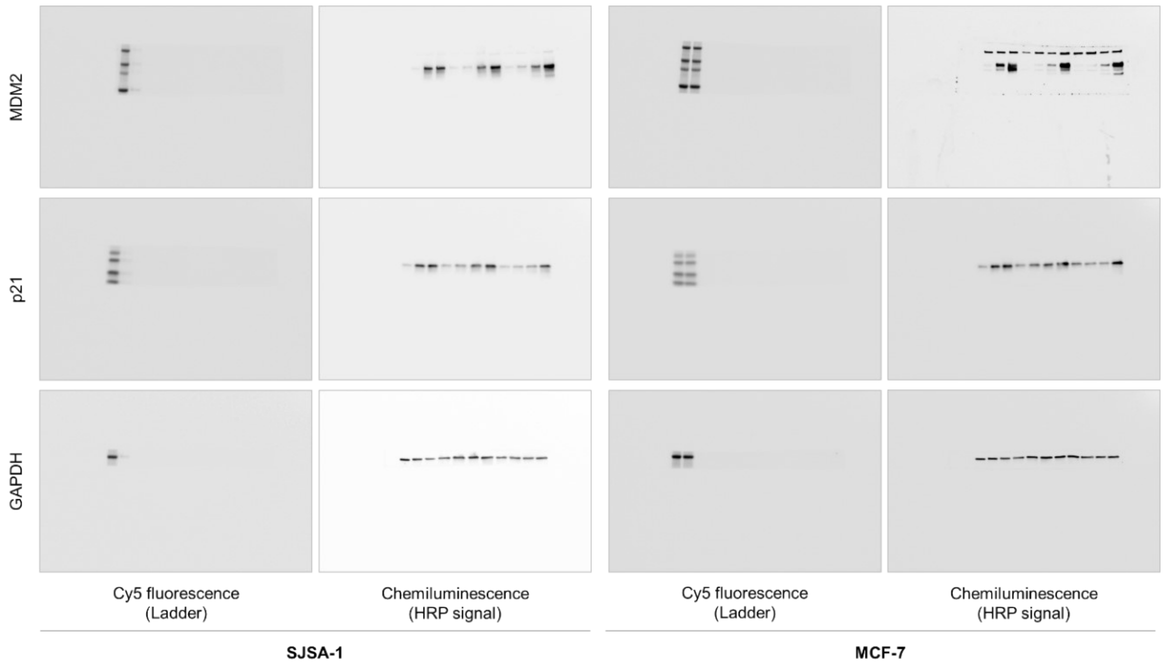

## Figure 3B

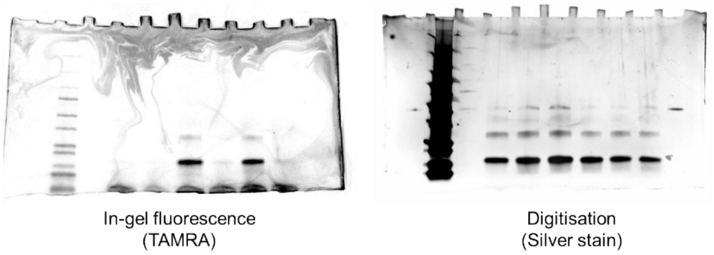


## Figure 3C and Figure S4

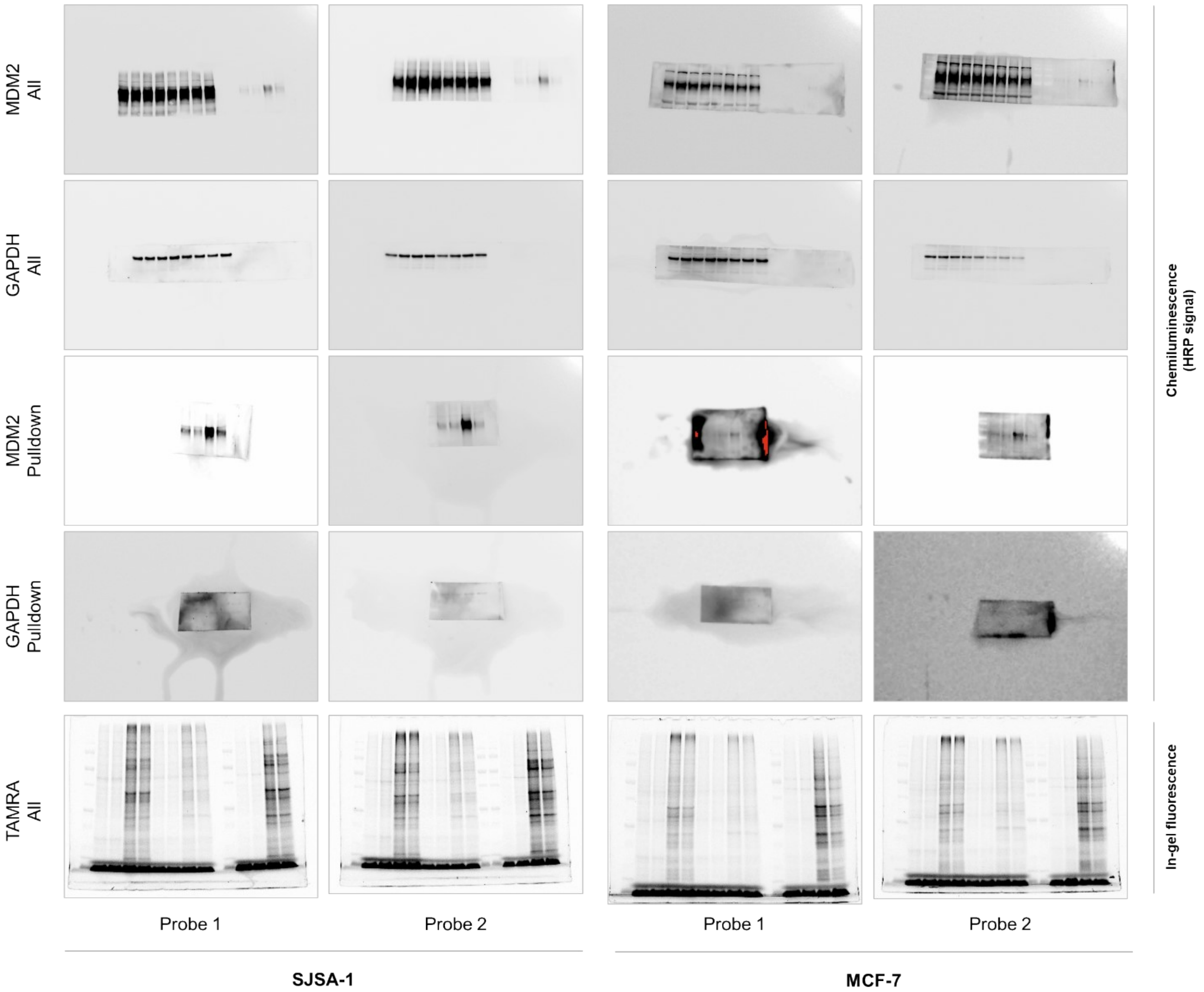

## Figure S2(B)

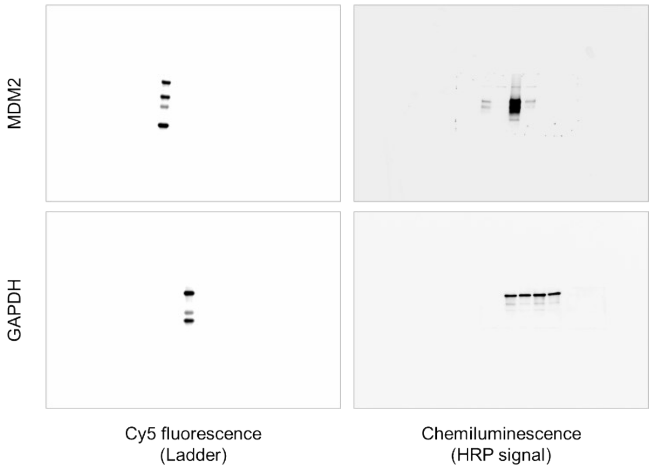


## Figure S3(C)

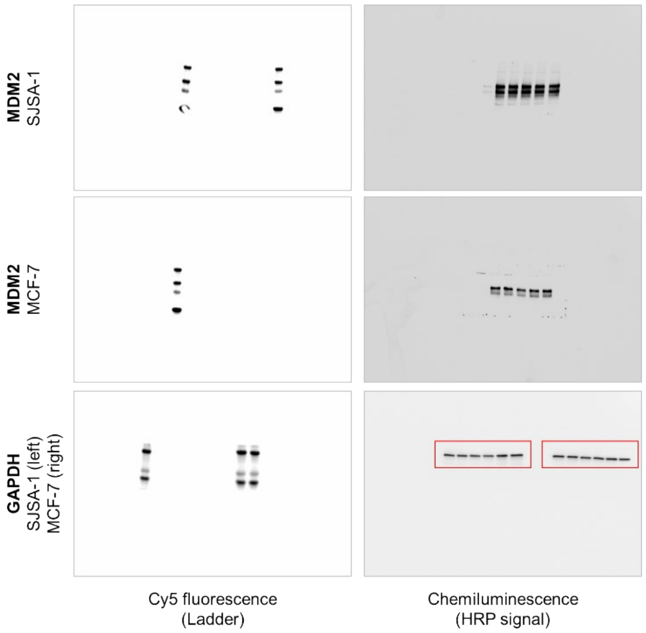

Supplement: SC-016-D5SC00120J-s001 [file SC-016-D5SC00120J-s001.pdf]
